# User-Centered Development of a Digital Health Service for Diabetic Foot Ulcer Risk Stratification: Usability Study

**DOI:** 10.2196/83287

**Published:** 2026-04-30

**Authors:** Ulla Hellstrand Tang, Shivani Ravichandran, Stefan Candefjord, Dipu T Sathyapalan, Vivek Lakshmanan

**Affiliations:** 1 Department of Prosthetics and Orthotics at the Department for Biomedical Engineering and Medical Physics, Sahlgrenska University Hospital, Falkenbergsgatan 3, Gothenburg, 41285, Sweden, 46 706397913, 46 31408162; 2 Department of Orthopaedics, Institute of Clinical Sciences, Sahlgrenska Academy, University of Gothenburg, Gothenburg, Sweden; 3 Department of Electrical Engineering, Chalmers University of Technology, Gothenburg, Sweden; 4 Division of Infectious Diseases, Amrita Institute of Medical Science and Research Centre, Department of General Medicine, Amrita Vishwa Vidyapeetham, Kochi, Kerala, India; 5 Department of Podiatry, Amrita Institute of Medical Science and Research Centre, Amrita Vishwa Vidyapeetham, Kochi, Kerala, India

**Keywords:** diabetic foot, diabetes mellitus, user-centered design, clinical decision support system, digital health

## Abstract

**Background:**

Globally, 537 million persons live with diabetes, and a lifetime risk of up to 34% of developing diabetic foot ulcers (DFUs) necessitates strengthened preventive initiatives.

**Objective:**

The study aimed to develop and evaluate a clinical decision support system (CDSS) to be used by health care professionals in foot assessment and risk stratification as a base for prevention.

**Methods:**

Based on principles of human-computer interaction, the CDSS was developed for DFU risk assessment. Users, health care professionals from Region Västra Götaland in Sweden, evaluated the functions regarding effectiveness, efficiency, and satisfaction using a mixed methods usability testing approach. Expectations and experiences of using the CDSS were evaluated with the System Usability Scale (SUS).

**Results:**

A total of 9 participants participated. User expectations of the CDSS, measured by SUS, averaged 77.2 (SD 14.6). Posttest SUS scores were 68.9 (SD 14.3), with a mean difference of 8.3 (*P*=.07), a nonsignificant reduction of usability after testing. The effectiveness of the CDSS in supporting users to complete 9 clinical tasks showed that for 7 (78%) tasks, at least 5 (56%) testers successfully achieved the intended goals. Tasks involving the identification of ingrown toenails and the confirmation of foot status, including risk stratification for the patient, were completed by fewer testers. Efficiency, measured as mean task completion time, ranged from 7 seconds to 9 minutes 20 seconds, and qualitative feedback informed recommendations for further system refinement. Users reported that a structured CDSS has the potential to support more equitable, consistent, and person-centered DFU prevention within a digital health service.

**Conclusions:**

A digital health service for DFU risk stratification was developed based on national and international guidelines. Although the users’ expectations of the usability were higher compared to how they experienced the CDSS, the SUS test was near a threshold of 70, indicating that the system being tested was above average in usability. Further development and validation, both nationally and internationally, with continued attention to users’ needs and contextual factors, are recommended.

## Introduction

### Background

**Figure 1.**
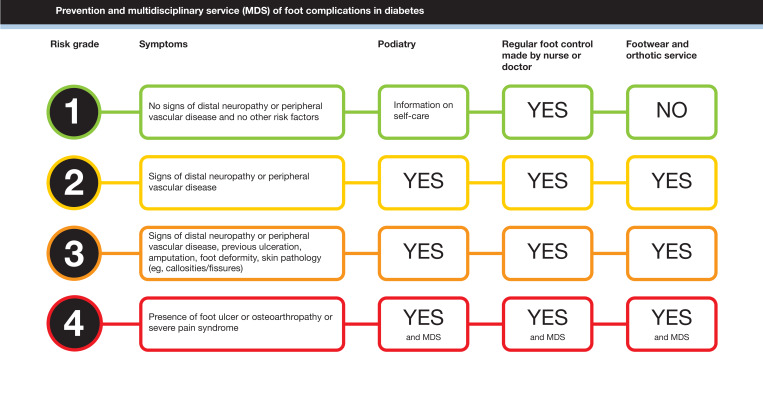
Schema of the risk stratification as described in Swedish national clinical guidelines [8].

Globally, 589 million persons live with diabetes [1], with a lifetime risk of developing diabetic foot ulcers (DFUs) ranging from 19% to 34% [2]. Out of 10.6 million people in Sweden, 600,000 persons had diabetes in 2024, according to the Swedish National Diabetes Register (NDR) [3]. In India, an increase from 90 million people with diabetes in 2024 to 157 million in 2050 is estimated [1]. DFUs are a serious and growing global health challenge. When left untreated or not promptly addressed, DFUs can lead to severe complications, including lower-extremity amputation, significantly affecting patients’ quality of life and increasing health care costs [4,5]. The 5-year mortality rate following DFU is 30% due to complications related to diabetes and comorbidities [6]. Worldwide, 18.6 million people live with DFU, with vascular, neurological, and biomechanical components [2]. The International Working Group on the Diabetic Foot identifies the following key risk factors for DFU development: peripheral neuropathy, peripheral vascular disease, skin pathologies, foot deformities, and a history of previous DFU or amputations [7] (Figure 1). These risk factors need to be identified regularly in each person living with diabetes. Despite well-established guidelines recommending regular foot examinations and risk assessments, a significant proportion of at-risk patients do not undergo structured screening for DFU risk in Swedish clinical practice. For instance, data from NDR indicate that 25% of patients in Sweden do not undergo the recommended foot examinations [3]. Timely interventions, such as podiatry and therapeutic footwear, may be omitted. Early prevention is recommended to hinder DFU progression and reduce the risk of amputation, and rapid referral to multidisciplinary care teams is recommended for patients diagnosed with DFUs [7].

### The Role of Digital Health Services in the Prevention of Foot Ulcers

The accelerated digitalization of health care has prompted efforts to integrate clinical decision support systems (CDSSs) [9] that allow health care professionals (HCPs) to register patient data, store it securely, and use it for evaluation and audit, and potentially for artificial intelligence (AI)–driven outcome prediction. However, despite efforts to develop digital health solutions, promoting foot health for patients with diabetes, no integrated system is currently in use in Sweden or elsewhere (as of 2025). On a global scale, Kabir and Ashmed [10], in their review, found that existing software for DFU management lacks sufficient evidence-based reliability, emphasizing the need for systematic evaluation. Their review of 170 applications identified major shortcomings, such as inadequate assessment tools, poor integration of best practices, and limited use of AI-driven enhancements. To ensure that these applications meet health care standards, a robust evaluation framework, such as human-computer interaction (HCI), is crucial, ultimately enhancing DFU management, improving patient outcomes, and reducing health care costs [10].

### Theoretical Framework: HCI

The field of HCI has evolved beyond its initial focus on computer-centric usability to encompass diverse technologies, including CDSS. HCI research aims to optimize interactions between users and digital tools. Bevan and Harker [11] contributed to improving human interaction design in health care by refining the ISO 9241‐11 usability standard [12], emphasizing a more comprehensive understanding of usability beyond efficiency, effectiveness, and satisfaction. User-centered design is an iterative development methodology that prioritizes user needs, tasks, and environmental factors throughout the design and implementation processes. This approach ensures that digital tools align with end users’ requirements, thereby enhancing adoption and clinical use. Furthermore, evaluating the usability of the system is crucial in order to ensure that the system fulfills the needs and performs effectively in clinical settings [13].

Grundgeiger et al [14] advocated for incorporating user experience (UX) principles in safety-critical health care environments. They emphasized that beyond usability, factors such as emotional response, trust, and cognitive workload significantly impact HCPs’ interactions with technology. UX refers to the overall impact of a user’s interaction with a system, spanning initial impressions, long-term engagement, and emotional responses. Usability, a core component of UX, refers to the effectiveness, efficiency, and satisfaction with which users achieve specified goals in a given context. The concept of “UX” by Norman et al [15] highlights the importance of a holistic interaction design beyond interface usability.

A limited number of applications, such as CONNECTPlus [16] and D-Foot [17-19], have been specifically developed for HCPs to support systematic identification of DFU risk and structured foot assessment. Both D-Foot and CONNECTPlus aim to facilitate risk stratification and support clinical decision-making by providing structured assessment tools and guidance.

D-Foot, developed within Region Västra Götaland (VGR) in western Sweden, was designed for use by certified prosthetists and orthotists. The system is based on a structured foot examination aligned with recommendations from the Swedish Association of Local Authorities and Regions [8,20].

CONNECTPlus offers features such as risk-specific education, self-management support, and remote monitoring intended to empower patients and potentially reduce health care burden; however, its direct impact on clinical outcomes has not yet been established.

Prior to this study (in 2020), the principal investigator (UT) led a regional development group in the VGR that, based on national guidelines [20], created a workflow for a foot examination in paper format to facilitate risk stratification for patients with diabetes (Multimedia Appendix 1). The face validity of the foot examination was secured by HCPs regionally and nationally during 2 workshops. The foot examination in paper format, with its structured foot assessment, has previously been explored regarding how HCPs experienced its use during foot assessments of patients with diabetes [17]. The HCPs found that the foot examination simplified examinations, highlighted differences in current standard management, and had the potential to standardize care and achieve good, equal, and person-centered care [17]. While some inconsistencies in risk categorization were noted, the anticipated digital version was expected to enhance documentation, accessibility, and adherence to guidelines.

### Aim and Research Question

Despite digital advancements, no CDSS is incorporated in a digital health service aimed to be used by HCPs, such as nurses, podiatrists, and doctors, for the early detection of risk factors that precede the development of DFUs. To address this gap, this study presents the design and evaluation of a digital health service including a CDSS for managing structured foot examinations.

The specific research questions were as follows:

1. How should a CDSS be designed to effectively capture and support the workflow in diabetic foot care?
2. Does the CDSS function as intended in meeting user expectations and supporting HCPs in real-world settings?
3. How does usability with pretest and posttest surveys contribute to refining the design of a CDSS?

## Methods

### Design and Setting

The study was part of a larger research initiative aimed at optimizing the prevention and care of patients with diabetes at risk of developing DFUs. The study was conducted in collaboration with VGR, the public health care provider for the second largest region in Sweden, serving approximately 1.8 million inhabitants. The study was carried out in 2 health care settings within VGR: Skaraborg Hospital in Skövde and Sahlgrenska University Hospital in Gothenburg (Multimedia Appendix 2). Usability testing sessions were conducted at these hospital sites during 2022. Each session lasted approximately 2 hours and was designed to allow in-depth observation and interaction with the CDSS in a controlled clinical environment. In addition, at a later stage, researchers from Amrita University in India were involved as a reference group.

The methodology comprised three phases—(1) design, (2) testing, and (3) iterative refinement—following established usability testing principles described by Dumas and Redish [21].

### Design Phase

In the design phase, the following steps were taken:

1. An innovation team, consisting of a clinical researcher, a researcher in health informatics, a master's student in biomedical engineering, and a researcher in biomedical engineering, formulated the strategy for the development of the digital health service.
2. The requirements for the digital health service were defined based on the foot examination that HCPs had created (Multimedia Appendix 1), a literature review, patients’ needs as expressed in the national clinical guidelines [8], and a review of existing diabetic foot care workflows in the VGR.
3. The key steps in diabetic foot care were identified and integrated into the design. Personas were created (Multimedia Appendix 3). Personas are useful in HCI and are fictional yet research-based representations of specific user groups, helping designers [22]. They guide design decisions by encapsulating user characteristics, behaviors, and goals, ensuring usability and accessibility in the development of interactive systems [23,24].
4. The software Lucidchart [25] (Multimedia Appendix 4), a web-based diagramming application, was used to create the conceptual design by visualizing the workflow anticipated in the digital health service during foot examination. The workflow for HCPs included useful features such as an education section for enhanced learning during use, an examination section, and a documentation section.
5. In the development of the digital health service, the software Figma (Dylan Field and Evan Wallace), a collaborative web application useful in interface design, was used (Multimedia Appendix 5) [26].

### The Test Phase

**Figure 2.**
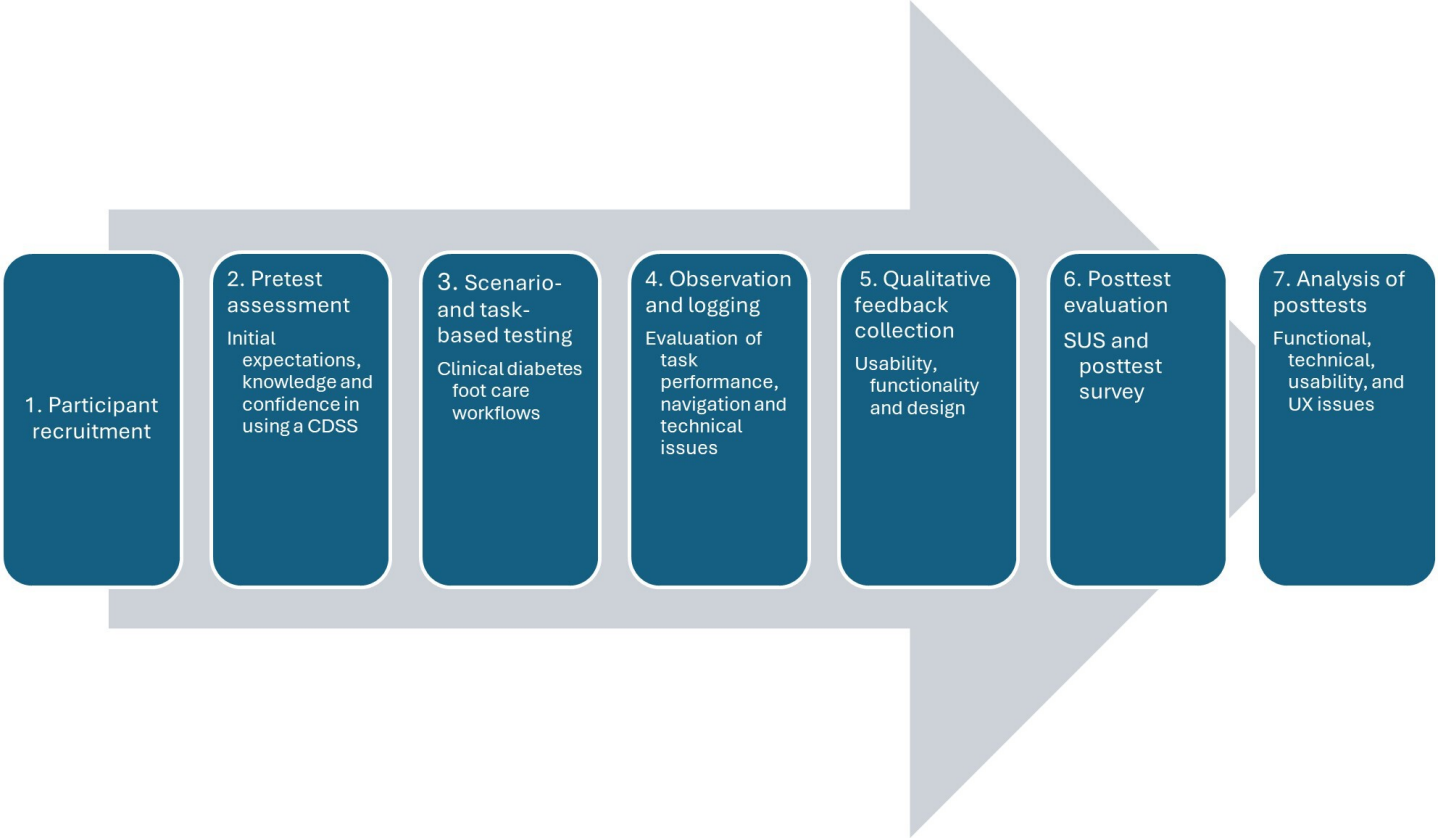
The test phases. CDSS: clinical decision support system; SUS: System Usability Scale; UX: user experience.

The usability testing followed a structured, sequential process (Figure 2). First, users’ initial expectations, knowledge, and confidence in using the CDSS were assessed using a pretest questionnaire (Multimedia Appendix 6). This was followed by scenario- and task-based testing, during which HCPs performed tasks reflecting routine diabetic foot care workflows. User interactions were observed to identify technical issues and assess whether functional requirements were met.

Qualitative feedback was then collected regarding usability, functionality, and design. Usability was further evaluated using pretest and posttest surveys, including the System Usability Scale (SUS), to assess user expectations and satisfaction [27-30]. Finally, posttest surveys (Multimedia Appendix 7) were used to assess users’ perceptions of how well the CDSS met their needs and expectations, as well as to identify issues related to ease of use, navigation, and overall UX.

### Participants

Participants were HCPs employed within VGR and were recruited using a purposive sampling approach. Inclusion criteria were that participants had clinical experience working with patients with diabetes at risk of developing DFUs. Eligible HCPs were systematically contacted by a member of the research team, informed about the study, and invited to participate in the usability testing.

A total of 9 HCPs participated in the study, including 3 nurses, 4 podiatrists, 1 physiotherapist, and 1 certified prosthetist and orthotist. Participants’ ages ranged from 37 to 68 years, and their clinical experience in diabetes care ranged from 1 to 40 years. To support detailed observation and interaction, each usability testing session included a maximum of 3 participants. The number of participants was judged to be sufficient, given the diversity of professions among testers and considering the cost of testing against the value of the information gained [31].

### Ethical Considerations

The study was approved by the Swedish Ethical Review Authority (register number 2020‐02715) and was conducted according to the ethical principles described in the Declaration of Helsinki, following the Code of Ethics of the World Medical Association for experiments involving humans [32]. The participants received oral and written information about the study, including information on their right to withdraw from the study at any time without explanation. Participants were included after providing written informed consent, and all participants agreed to be photographed. Written informed consent was also obtained for the publication of any potentially identifiable images or data included in this article. There is always a risk of intrusion of integrity when personal experiences are shared with others. However, the advantages for future patients were judged to balance the possible inconvenience that participants might experience, for example, the time they spent on travel and participation in the study. The participants were ensured confidentiality and were informed that any concerns could be clarified by contacting the research team. No such concerns were expressed. The ClincalTrials.gov ID is NCT05692778.

Written informed consent was obtained from all individuals depicted in figures for publication of their images in this open access article. Privacy measures were implemented to protect participants. No personally identifying information (eg, names or other identifiers) is included in the image or accompanying text. The images are used solely for illustrative purposes within the context of the study. Participant data were de-identified prior to analysis, and no directly identifiable information (eg, names or personal identifiers) was included in the dataset. No such concerns were expressed. No compensation was provided to the individuals.

### Workshop for Usability Testing

The usability test was held in the Skaraborg region, at Skövde Hospital and in Gothenburg during 2022. First, during the workshop, the study participants filled in a survey capturing demographic data, professional background, and experience of using digital tools in assessing foot status in patients with diabetes. Participants answered a mix of closed-ended (yes/no, multiple choice) and open-ended questions with pretest surveys (Multimedia Appendix 6). Furthermore, the HCPs’ prior experiences and their expectations of working with digital tools were registered. During the tests, the participants performed simulated user tasks, and the usability was evaluated with the following measures: (1) the time it took to perform the tasks and (2) registration in a think-aloud protocol, meaning that users’ thoughts were registered while performing tasks [33].

None of the 9 testers had, at the time of the workshop, previously used digital tools for assessing foot status in patients with diabetes. Six testers had not previously used digital tools for assessing patients’ health either. However, 2 testers had registered health status in the NDR [3], and 1 tester had documented foot status of the patient with a photo stored in software, Piscara [34]. Eight of the 9 testers took photos of the patient’s foot during examination in clinical practice but without registering where the photos were stored. Five of the testers preferred to use iPad [35], 3 testers used Android systems (eg, Samsung, Xiaomi, OnePlus, and Motorola), and 1 tester did not know which system they used.

**Figure 3.**
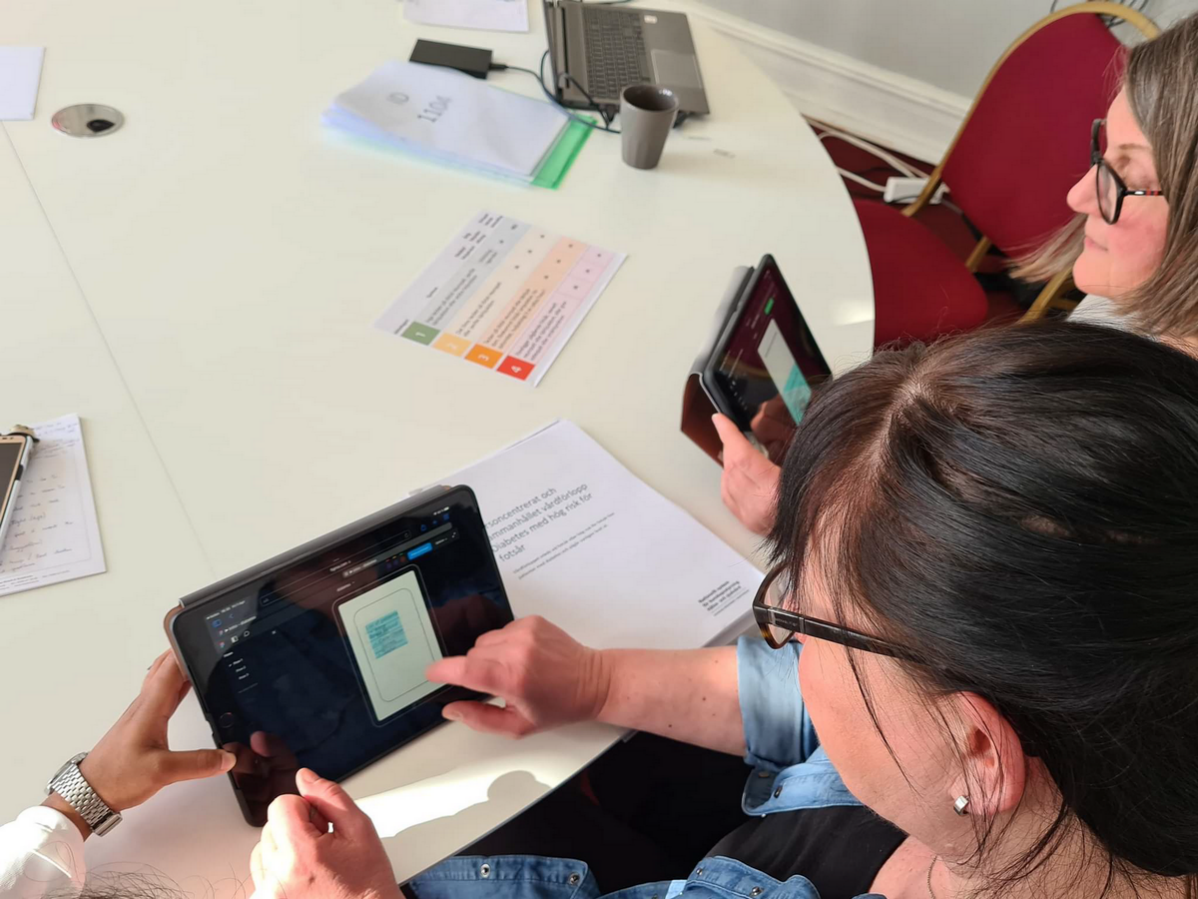
Usability testing of the digital health service. The testers were provided with tablets and documents in paper format with information about the usability test and the pretest and posttest surveys, and instructions on how to perform the usability tasks.

The usability test was introduced by 2 persons from the study team (SR and UT). The observers (n=2) made observations during the tests and registered comments from the testers, according to the think-aloud method [33]. The test sessions were recorded to be able to recall the comments made. The rooms used for the usability test were arranged so that testers had all the necessary equipment and information at hand (Figure 3), for example, a file, in paper format, was assigned to each of the testers with all instructions and the pretest and posttest surveys.

The English language was used, since the study was a collaboration with English-speaking researchers at Chalmers University of Technology and Amrita Vishwa Vidyapeetham, Kochi, in India. During the tests, the testers were free to ask questions in Swedish for clarification, and think-aloud comments were made both in English and in Swedish.

### Statistics

The study used a mixed methods design, combining qualitative and quantitative methods [36]. Quantitative data were analyzed using descriptive statistics and hypothesis testing. Participant characteristics were summarized using counts and percentages for categorical variables and means (SDs) for continuous variables. The SUS consists of 10 items rated on a 5-point Likert scale. Although individual items are ordinal, the SUS total score is calculated according to standard procedures to yield a composite score ranging from 0 to 100. Consistent with established practice, the aggregated SUS score was treated as continuous data and analyzed using parametric statistical methods, including paired Student *t* tests, to compare pretest and posttest scores [37], with a significance threshold set at *P*<.05. Task efficiency was assessed by measuring the time required to complete each predefined task and is reported as mean values with SDs. Task effectiveness was evaluated by calculating the proportion of participants who successfully completed each task.

Qualitative data from think-aloud protocols, observational notes, and open-ended survey responses were analyzed using an inductive content analysis approach supported by AI-assisted categorization. The AI tool was used to facilitate initial identification and grouping of meaning units in a data-driven manner. All preliminary codes and categories were subsequently reviewed, refined, and validated by the research team to ensure they accurately reflected the original data and study context. Final categorizations and interpretations were made by the researchers.

The study was reported in accordance with the CONSORT (Consolidated Standards of Reporting Trials) eHealth checklist (Checklist 1).

## Results

User expectations of the digital health service, measured with the SUS pretest, were 77.2 (SD 14.6), and the posttest SUS scores were 68.9 (SD 14.3), with a mean difference of 8.3 (*P*=.07), indicating a nonsignificant reduction in perceived usability after use. The effectiveness of the digital health services in supporting users to complete 9 specified clinical tasks showed that for 7 (78%) tasks, at least 5 (56%) testers successfully achieved the intended goals (Table 1). Tasks involving the identification of ingrown toenails and confirmation of foot status and risk stratification for a fictitious patient were completed by fewer testers. Participants expressed that a structured CDSS has the potential to contribute to more equitable and person-centered DFU prevention. Efficiency, measured as mean task completion time, ranged from 7 seconds to 9 minutes 20 seconds. The final task was to discuss the care plan with the fictitious patient, based on the risk stratification and foot status. One of the testers suggested that the foot assessment and the risk grade should be printed as a base for further discussion regarding self-care and care. In addition, it was suggested that the need for podiatry and the use of appropriate shoes should be discussed with the patients. Furthermore, it was suggested that the CDSS should automatically allow sending digital referrals for podiatry, to the Department of Prosthetics and Orthotics, and to specialist care.

**Table 1.** Effectiveness and efficiency of the usability tests (n=9).^a^

Task and specification of goals to reach	Effectiveness (goal reached)			Efficiency, mean (SD), mm:ss
	Yes, n (%)	No, n (%)	Missing, n (%)	
Warm-up task				
To enter the app	N/A^b^	N/A	N/A	05:15 (05:15)
Warm-up task				
To select your patient	N/A	N/A	N/A	00:23 (00:16)
Examine for pressure area				
Did the testers save Yes (pressure) for the left and right foot?	6 (67)	0 (0)	3 (33)	Missing
Did the testers examine both feet?	7 (78)	0 (0)	2 (22)	Missing
Examine for ingrown nails				
Did the testers save Yes (ingrown nails) for the right foot?	4 (44)	1 (11)	4 (44)	02:06 (01:13)
Examine for hallux valgus				
Did the testers click on the “Learn about” button?	5 (56)	3 (33)	1 (11)	00:37 (00:31)
Did the testers save Yes (hallux valgus) for the right foot?	7 (78)	1 (11)	1 (11)	—^c^
Ask the fictitious patient if he has peripheral neuropathy				
Did the testers save “Loss of sensation” for the left and the right foot?	6 (67)	1 (11)	2 (22)	02:39 (02:39)
Examine the fictive patient to check if he has peripheral neuropathy				
Did the testers click on the “Learn about” button?	7 (78)	1 (11)	1 (11)	03:38 (02:42)
Did the testers save “Loss” for the left and the right foot	7 (78)	0 (0)	2 (22)	—
Look at the summary and confirm that results are “OK”				
Look at the summary and confirm that results are “OK”	0 (0)	0 (0)	9 (100)	00:48 (01:12)
Discuss the care plan with the fictitious patient				
Did the testers show the results to the fictitious patient?	6 (67)	0 (0)	3 (33)	02:04 (01:48)
What discussion did the nurse/tester have with the fictive patient regarding the care plan?	1 (11)	0 (0)	8 (89)	—

a This table summarizes the results of the usability testing in terms of effectiveness, efficiency, and task completion metrics. Nine main tasks were included. In the column “Specification“ column, details about each task are presented. Efficiency, meaning the time spent to execute the tasks, is presented in the “Efficiency” column.

b N/A: not applicable.

c Not available.

Several useful suggestions for refining the design of the CDSS were recorded, based on the think-aloud protocols. The following themes were identified: (1) referrals and communication, (2) foot examination features, (3) user interface and navigation, (4) training and usability, (5) technical issues, (6) workflow and click analysis, (7) content feedback, and (8) login and simplicity (Table 2).

Results from the posttest survey showed that all testers, except 1, would prefer to use a tablet in future digital foot examinations, with the rationale that the screen is larger compared with a smartphone screen. For 1 tester, the type of hardware did not matter. All testers would prefer an automatically generated risk scale in the final CDSS, commenting that an automatically generated risk scale could enhance clarity and flexibility and improve security regarding care. One comment was “Becomes clear and allows you to assess and inform about the result,” and there were comments that the assessment made by the HCP of the overall need of each patient is necessary, assuring person-centered care. A total of 8 (89%) participants found it useful to register foot status for the left and the right foot separately and gave the rationale that it is of importance to address separate registration, thereby ensuring clarity and distinction. Instead of the current design, involving clicking forwards and backwards in the CDSS, 5 (56%) participants would prefer a scroll-down feature to navigate between questions. The structure of the CDSS was described as logical, clear, and good by 4 (44%) of the participants. A minor challenge noted was that it was “somewhat complicated, especially the language,” as English was used instead of Swedish.

**Table 2.** Suggestions for improvements of the digital health service.

Theme	Feedback and comments
Referrals and communication	Enable digital referrals to podiatry and prosthetics and orthotics based on the foot summary. Provide an option to print or send a referral link to the patient’s mobile. Patients should be able to independently contact specialists (in person, by phone, or by email).
Foot examination features	Ability to take and store photos of the feet (dorsal and plantar views). Ensure image access for both patients and health care personnel.
User interface and navigation	Include a “Back” button or icon on all question pages. Allow zoom-in functionality for images. Add a search function for the patient list. Highlight “Yes/No” buttons upon selection for validation. Consider removing the “Save” button if selection highlights clearly indicate that input has been registered. Add visual confirmation (eg, click animation) to indicate when buttons are pressed. Clarify whether all questions must be answered by the HCP^a^.
Training and usability	Provide user training prior to access. Show tester feedback below each question. Clarify whether illustrations (eg, ingrown nails) depict real cases or demos. Address communication barriers between testers and observers.
Technical issues	Device required firm pressure due to the screen protector. Device performed better when handheld rather than lying flat. “Learn about” button was overlooked during testing.
Workflow and click analysis	Task 3 required 15 clicks to access the page and complete the task (tested 3 times). Task 4 required 7 clicks before returning to “Back to inspection.” Suggest reducing the total number of clicks to simplify navigation.
Content feedback	Ingrown toenail assessment was misinterpreted as evaluating thickened nails. Illustrations need clearer contextual explanations.
Login and simplicity	Request for a simpler login process.

a HCP: health care professional.

## Discussion

### Principal Findings

This study aimed to design and evaluate a digital health service to support HCPs in conducting structured foot assessments and risk stratification for DFUs. The principal findings indicate that the developed CDSS was usable and acceptable to HCPs across diverse professional backgrounds and varying levels of digital experience. Most participants were able to complete the majority of predefined clinical tasks, and the system demonstrated reasonable effectiveness and efficiency in supporting structured DFU assessment workflows. Although posttest usability ratings were slightly lower than pretest expectations, overall usability remained close to established acceptability thresholds. Importantly, participants perceived that a structured CDSS could support more consistent, equitable, and person-centered DFU prevention, aligning with the overarching objective of improving early detection and preventive care [38].

The usability and acceptance of the CDSS observed in this study are consistent with previous research indicating that structured foot assessment and digital decision support tools can assist HCPs in standardizing DFU assessments and improving adherence to clinical guidelines [38-42]. Similar to earlier usability studies of digital health interventions, participants were able to engage with the system despite limited prior experience with comparable tools, underscoring the importance of intuitive design and workflow alignment in early-stage implementations [43].

The observed decrease in SUS scores from pretest to posttest, although not statistically significant, reflects a pattern reported in other usability studies, where initial expectations tend to exceed postuse perceptions once users encounter practical constraints and design limitations [29,44]. This underscores the importance of formative usability testing in identifying mismatches between user expectations and real-world system performance. Tasks related to identifying specific foot conditions and confirming final risk stratification were less consistently completed, suggesting that these cognitively demanding steps require clearer visualization and decision support. Previous studies on DFU risk assessment have similarly reported challenges in ensuring consistent interpretation of clinical findings, particularly when multiple risk factors must be integrated into a final classification [45]. Participants’ emphasis on automated risk scoring and clearer differentiation between left and right foot assessments aligns with literature advocating for cognitive support features to reduce variability and enhance clinical decision-making [46]. Beyond usability, the perception that the CDSS could support person-centered and equitable care is in line with growing evidence that structured digital tools can improve communication, transparency, and shared understanding between HCPs and patients in chronic disease management [47].

### Usability and Acceptance of the CDSS

The CDSS was generally well received by participating HCPs, representing varied professional backgrounds and clinical experience. Notably, although the majority of participants had little or no prior experience of using digital health services for diabetic foot assessments, they were able to engage with the current CDSS and complete most of the clinical tasks. Pretest SUS scores indicated high expectations (77.2, SD 14.6), while posttest scores were slightly lower (68.9, SD 14.3), though the difference was not statistically significant. This reduction suggests that while the tool was functional and promising, aspects of its usability require improvement to better meet user expectations.

Several usability challenges were identified through think-aloud protocols and posttest surveys, including different navigation options (eg, scroll-down vs page-by-page design), difficulties with English-language prompts, and requests for clearer supporting information. These findings underline the importance of an iterative user-centered design and the value of including real users in early testing stages to refine the tool for real-world use [12].

### Effectiveness in Supporting Clinical Tasks and Workflow

The CDSS demonstrated reasonable effectiveness in supporting users through structured foot assessment workflows. Fifty-six percent of the participants completed 7 of 9 clinical tasks successfully, showing that the tool facilitated critical steps in DFU risk evaluation. However, tasks involving the identification of specific conditions (eg, ingrown toenails) and final risk stratification were less consistently completed, pointing to areas where the interface or decision logic could be enhanced. Participants emphasized the importance of an automatically generated risk score, separate documentation for the left and the right foot, and real-time feedback during the assessment. These suggestions align with the goal of supporting clinical reasoning and improving consistency in risk classification, which is vital for ensuring timely and accurate referrals and interventions. Importantly, the tool was perceived to promote equitable and person-centered care, a critical aspect of DFU prevention in both high-resource and resource-constrained settings [48].

### Implications for Future Development and Implementation

Despite the growing digitalization in health care, few digital tools currently exist that are tailored for early DFU detection by HCPs. This study addressed a clear gap by developing a CDSS grounded in national guidelines, evidence-based practice, and real-world workflows. The digital health service has the potential to be scaled and localized for broader use, particularly given the global burden of diabetes and DFUs in Sweden and India, 2 countries represented in this project.

For successful implementation, future iterations of the CDSS should incorporate language adaptation, enhanced onboarding and training modules, and seamless integration with existing electronic health records (EHRs) or quality registers such as the Swedish NDR [3]. Furthermore, expanding tests to include patients and HCPs in clinical settings will be essential to validating the CDSS’s effectiveness in reducing DFU incidence and improving patient outcomes.

To facilitate the usability test, the tests were conducted close to where the HCPs worked, in Skövde and Gothenburg, respectively. It was considered easier to perform the test on site where the HCPs worked, as they did not need to travel to a usability lab situated elsewhere. This arrangement allowed HCPs working in the city of Gothenburg in the Skaraborg region, that is, in the countryside, respectively, to contribute with their experiences of using the CDSS.

### Methodological Considerations

A strength of this study was the integration of qualitative and quantitative data within a mixed methods framework. Findings from think-aloud sessions and user observations were triangulated with survey responses to identify convergent usability issues and inform iterative refinement of the CDSS. This integration enabled the translation of user feedback into concrete design improvements, including enhanced navigation functions and refinement of the output report to support patient communication and referral decisions. Another strength of the study was the diversity among the testers in professional backgrounds, experiences, and familiarity with technology.

A limitation of this study is the small sample size for the usability evaluation (n=9), which may be considered low from a research perspective. However, this number is consistent with established methodological guidance for formative usability testing in user-centered design, where the primary aim is to identify usability issues rather than to achieve statistical generalizability. Prior work demonstrates that most usability problems are detected with small samples and that returns diminish beyond approximately 5 to 10 participants [49-51]. Nevertheless, the findings should be interpreted as indicative rather than definitive, and additional usability issues may emerge with broader testing. Another limitation is that the sample may not fully capture the heterogeneity of intended end users, including variations in clinical settings, digital literacy, and demographic characteristics. In addition, a survey question regarding prior use of digital tools may have been misinterpreted, as participants did not consistently consider routine use of EHRs as use of a digital tool. The mixed use of English and Swedish during usability testing also constituted a limitation, as it may have contributed to uncertainty during task performance and missing data. Furthermore, some visual elements and task formulations led to misinterpretation (eg, illustration of an ingrown toenail as a thickened toenail), indicating areas requiring refinement in future iterations of the CDSS.

Finally, a limitation is that implementation was not examined as an explicit object of inquiry using a formal implementation framework. Although organizational, contextual, and workflow-related issues emerged during usability testing, these were not systematically analyzed using an implementation science framework, such as the Nonadoption, Abandonment, Scale-up, Spread, and Sustainability Clinical Assessment Tool [52]. Previous work from the western region of Sweden has highlighted complex challenges related to technical readiness and organizational preparedness for the development and implementation of CDSSs [53]. Consequently, determinants related to long-term adoption, scale-up, and sustainability in real-world clinical settings were not comprehensively assessed in the present study and should therefore be interpreted with caution.

### Future Development

Future work should include summative usability evaluations and real-world implementation studies involving larger and more diverse user groups to assess generalizability, clinical effectiveness, and impact on patient outcomes. Further development must also align with regulatory requirements for medical devices, particularly the European Medical Device Regulation, including clinical validation, usability engineering, and risk management [54]. Beyond usability, future studies should evaluate the accuracy, reliability, and adaptability of automated risk stratification algorithms while ensuring that clinicians retain the ability to exercise clinical judgment. Integration with EHRs and quality registers, as well as structured onboarding and training for HCPs, will be essential for sustainable implementation. To support broader adoption, future research should explore cultural and regional adaptations of the CDSS, including use in resource-constrained settings, hardware preferences (eg, tablet vs smartphone), and workflow integration across different health care systems. Addressing these factors will be essential to realizing the full potential of digital decision support in advancing equitable and person-centered DFU prevention.

This study demonstrates the feasibility of developing a user-centered CDSS to facilitate structured foot assessment and DFU risk stratification in clinical practice. The findings indicate that the CDSS was usable and acceptable to HCPs with diverse backgrounds and levels of digital experience, and that it can support consistent, efficient, and person-centered preventive workflows. While further development, validation, and implementation-focused research are required, this work provides important formative evidence and design insights that can inform future digital decision support solutions aimed at improving early detection and prevention of diabetic foot complications.

## Supplementary material

10.2196/83287 Multimedia Appendix 1 Structured foot examination.

10.2196/83287 Multimedia Appendix 2 Skaraborg and Gothenburg in the Region Västra Götaland [55].

10.2196/83287 Multimedia Appendix 3 Personas a fictive person living with diabetes and a foot ulcer.

10.2196/83287 Multimedia Appendix 4 Workflow development for the digital health service using Lucidchart.

10.2196/83287 Multimedia Appendix 5 Selected screenshots of clinical decision support system workflow evaluated in the workshop.

10.2196/83287 Multimedia Appendix 6 Pretest survey.

10.2196/83287 Multimedia Appendix 7 Posttest survey.

10.2196/83287 Checklist 1 CONSORT-eHEALTH (V1.6.1) checklist.

## Abbreviations

AI: artificial intelligence
CDSS: clinical decision support system
CONSORT: Consolidated Standards of Reporting Trials
DFU: diabetic foot ulcer
EHR: electronic health record
HCI: human-computer interaction
HCP: health care professional
NDR: National Diabetes Register
SUS: System Usability Scale
UX: user experience
VGR: Region Västra Götaland

## References

[1] (2025). IDF diabetes atlas 2025. https://diabetesatlas.org/resources/idf-diabetes-atlas-2025/.

[2] Armstrong DG, Tan TW, Boulton AJM, Bus SA (2023). Diabetic foot ulcers: a review. JAMA.

[3] (2025). Nationella Diabetesregistret [Website in Swedish].

[4] Edmonds M, Manu C, Vas P (2021). The current burden of diabetic foot disease. J Clin Orthop Trauma.

[5] McDermott K, Fang M, Boulton AJM, Selvin E, Hicks CW (2023). Etiology, epidemiology, and disparities in the burden of diabetic foot ulcers. Diabetes Care.

[6] Armstrong DG, Swerdlow MA, Armstrong AA, Conte MS, Padula WV, Bus SA (2020). Five year mortality and direct costs of care for people with diabetic foot complications are comparable to cancer. J Foot Ankle Res.

[7] Schaper NC, van Netten JJ, Apelqvist J (2024). Practical guidelines on the prevention and management of diabetes-related foot disease (IWGDF 2023 update). Diabetes Metab Res Rev.

[8] (2022). Personcentrerat och sammanhållet vårdförlopp diabetes med hög risk för fotsår [Report in Swedish]. https://d2flujgsl7escs.cloudfront.net/external/Vardforlopp_Diabetes.pdf.

[9] Sutton RT, Pincock D, Baumgart DC, Sadowski DC, Fedorak RN, Kroeker KI (2020). An overview of clinical decision support systems: benefits, risks, and strategies for success. NPJ Digit Med.

[10] Kabir A, Ahmed S Future of foot care: mobile apps in diabetic ulcer management. https://researchoutput.csu.edu.au/en/publications/future-of-foot-care-mobile-apps-in-diabetic-ulcer-management/.

[11] Bevan N, Harker S ISO 9241-11 revised: what have we learnt about usability since 1998?.

[12] (2019). ISO 9241-210:2019 ergonomics of human-system interaction—part 210: human-centred design for interactive systems. https://cdn.standards.iteh.ai/samples/77520/8cac787a9e1549e1a7ffa0171dfa33e0/ISO-9241-210-2019.pdf.

[13] (2012). Innovating for People: Handbook of Human-Centered Design Methods: Luma Institute.

[14] Grundgeiger T, Hurtienne J, Happel O (2021). Why and how to approach user experience in safety-critical domains: the example of health care. Hum Factors.

[15] Norman DA, Miller J, Henderson A What you see, some of what’s in the future, and how we go about doing it: HI at apple computer. https://www.researchgate.net/publication/202165701_What_You_See_Some_of_What’s_in_the_Future_And_How_We_Go_About_Doing_It_HI_at_Apple_Computer.

[16] (2025). CONNECTPlus: reducing the risk of diabetic foot ulceration. Health and Care Innovations Limited.

[17] Andersson S, Scandurra I, Nyström U, Varemo M, Tang UH (2023). Experiences of a novel structured foot examination form for patients with diabetes from the perspective of health care professionals: qualitative study. JMIR Nurs.

[18] Hellstrand Tang U, Tranberg R, Sundberg L, Scandurra I (2024). How do patients and healthcare professionals experience foot examinations in diabetes care? - A randomised controlled study of digital foot examinations versus traditional foot examinations. BMC Health Serv Res.

[19] Hellstrand Tang U, Tranberg R, Zügner R (2017). The D-Foot, for prosthetists and orthotists, a new eHealth tool useful in useful in risk classification and foot assessment in diabetes. Foot Ankle Online J.

[20] Östensson CG, Johansson K, Insulander L, Jonsson LV, Löndahl M, Sergu-Bogdan C (2018). Fotundersökning vid diabetes-nationellt vårdprogram för prevention av fotkomplikationer vid diabetes [Report in Swedish]. https://swenurse.se/download/18.32612fa517b3be241ddb892d/1629654183409/fotundersokning-vid-diabetes.pdf.

[21] Dumas JS, Redish JC (1999). A Practical Guide to Usability Testing.

[22] Adlin T, Pruitt J, Goodwin K Putting personas to work.

[23] Reimann R, Cooper A, Cronin D, Noessel C (2014). About Face: The Essentials of Interaction Design.

[24] Soegaard M (2018). The basics of user experience design. https://bpb-eu-w2.wpmucdn.com/sites.aub.edu.lb/dist/c/13/files/2019/06/the-basics-of-ux-design.pdf.

[25] (2025). Lucidchart.

[26] (2025). Figma.

[27] Brooke J (1996). Usability Evaluation in Industry.

[28] Brooke J (2013). SUS: a retrospective. J Usability Stud.

[29] Bangor A, Kortum PT, Miller JT (2008). An empirical evaluation of the system usability scale. Int J Hum –Comput Interact.

[30] Lewis JR, Sauro J (2018). Item benchmarks for the system usability scale. J Usability Stud.

[31] Borsci S, Macredie RD, Martin JL, Young T (2014). How many testers are needed to assure the usability of medical devices?. Expert Rev Med Devices.

[32] World Medical Association (2025). World Medical Association Declaration of Helsinki: ethical principles for medical research involving human participants. JAMA.

[33] Virzi RA, Sorce JF, Herbert LB (1993). A comparison of three usability evaluation methods: heuristic, think-aloud, and performance testing. Proc Hum Factors Ergon Soc Annu Meet.

[34] (2025). Omda picsara. Omda.

[35] (2025). Apple.

[36] Morse JM, Niehaus L (2016). Mixed Method Design: Principles and Procedures.

[37] Bangor A, Kortum P, Miller J (2009). Determining what individual SUS scores mean: adding an adjective rating scale. J Usability Stud.

[38] Bus SA, Sacco ICN, Monteiro-Soares M (2024). Guidelines on the prevention of foot ulcers in persons with diabetes (IWGDF 2023 update). Diabetes Metab Res Rev.

[39] Garces TS, Araújo Ad, Sousa GJB (2024). Clinical decision support systems for diabetic foot ulcers: a scoping review. Rev Esc Enferm USP.

[40] Zügner R, Jarl G, Sundberg L, Hellstrand Tang U (2022). Experiences of using a digital tool, the D-foot, in the screening of risk factors for diabetic foot ulcers. J Foot Ankle Res.

[41] Kumbhar S, Bhatia M (2024). Advancements and best practices in diabetic foot care: a comprehensive review of global progress. Diabetes Res Clin Pract.

[42] de Oliveira TCD, de Oliveira AF, de Castro Araújo L (2025). Digital health technologies for diabetic foot ulcers: a systematic review of clinical evidence, access inequities, and public health integration. Int J Environ Res Public Health.

[43] Kushniruk AW, Patel VL (2004). Cognitive and usability engineering methods for the evaluation of clinical information systems. J Biomed Inform.

[44] Lewis JR (2018). The System Usability Scale: past, present, and future. Int J Hum Comput Interact.

[45] Hidalgo-Ruiz S, Ramírez-Durán MDV, Basilio-Fernández B (2023). Assessment of diabetic foot prevention by nurses. Nurs Rep.

[46] Khairat S, Karar A, Khairat S (2025). Facilitators and barriers to ICU providers’ usability of electronic health records screens. Stud Health Technol Inform.

[47] Bitomsky L, Pfitzer E, Nißen M, Kowatsch T (2025). Advancing health equity and the role of digital health technologies: a scoping review. BMJ Open.

[48] Brousseau-Foley M, Blanchette V, Houle J, Trudeau F (2024). Developing an interprofessional decision support tool for diabetic foot ulcers management in primary care within the family medicine group model: a Delphi study in Canada. BMC Prim Care.

[49] Nielsen J, Landauer TK A mathematical model of the finding of usability problems. https://www.semanticscholar.org/paper/A-mathematical-model-of-the-finding-of-usability-Nielsen-Landauer/245061d94c228abdc9bda2d9a10679e897526465.

[50] Nielsen J (1999). Designing Web Usability: The Practice of Simplicity.

[51] Faulkner L (2003). Beyond the five-user assumption: benefits of increased sample sizes in usability testing. Behav Res Methods Instrum Comput.

[52] Greenhalgh T, Wherton J, Papoutsi C (2017). Beyond adoption: a new framework for theorizing and evaluating nonadoption, abandonment, and challenges to the scale-up, spread, and sustainability of health and care technologies. J Med Internet Res.

[53] Hellstrand Tang U, Smith F, Karilampi UL, Gremyr A (2024). Exploring the role of complexity in health care technology bottom-up innovations: multiple-case study using the nonadoption, abandonment, scale-up, spread, and sustainability complexity assessment tool. JMIR Hum Factors.

[54] Regulation (EU) 2017/745 of the European Parliament and of the Council of 5 April 2017 on medical devices. EUR-Lex.

[55] (2025). Region Västra Götaland [Article in Swedish].

