# Supplementary material for: User-Centered Development of a Digital Health Service for Diabetic Foot Ulcer Risk Stratification: Usability Study
Source: JMIR Diabetes. 2026 Apr 30;11:e83287. doi: 10.2196/83287 (PMC13132532; doi:10.2196/83287)
Supplement: Multimedia Appendix 1 [file diabetes-v11-e83287-s001.docx]

## Appendix 1. Structured foot examination

This form outlines the structured foot examination used in the study, detailing risk categories and associated clinical signs for both the right and left foot.

| Category | Clinical Sign/Question | Right Foot (R) | Left Foot (L) | Risk Level if "Yes" |
| --- | --- | --- | --- | --- |
| Examination and visual Inspection | Are there any ulcers? |  |  | 4 |
|  | Are there pressure sores, cracks, callosities, corns, or nail problems that could result in ulcer formation? |  |  | 3 |
|  | Is there dry skin, pale skin, altered temperature, swelling, or reduced hair growth? |  |  | 2 |
|  | Is there any toe/foot amputation, hallux valgus, or hammer toes? |  |  | 3 |
| Examination and palpation | Are the dorsalis pedis and/or tibialis posterior pulses non-palpable (if so, continue with Doppler exam)? |  |  | 2 |
|  | Is superficial nerve function reduced according to the Ipswich Touch Test or monofilament testing? |  |  | 2 |
|  | Is deep nerve function reduced, tested with a 128 Hz tuning fork? |  |  | 2 |
| Patient history and symptoms | Does the patient experience tingling, numbness, walking on pillows, or sensory changes in their feet? |  |  | 2 |
|  | Has the patient had a foot ulcer that was difficult to heal? |  |  | 3 |

## Risk Categorisation

Based on the clinical examination and patient history:
- Assign a risk level (1–4), with:
 - 1 = Healthy foot
 - 4 = Highest risk (e.g., current ulceration)

Instructions:
- Record the highest individual risk level observed.
- Document the overall risk category (1–4).
- Ensure that the highest risk level is reported to the National Diabetes Register (NDR).
- Confirm that the patient has received verbal and written self-care advice.
- Document all findings and actions in the medical record.
