# Supplementary figures and images for: User-Centered Development of a Digital Health Service for Diabetic Foot Ulcer Risk Stratification: Usability Study
Source: JMIR Diabetes. 2026 Apr 30;11:e83287. doi: 10.2196/83287 (PMC13132532; doi:10.2196/83287)

Appendix 2. Skaraborg and Gothenburg in the Region Västra Götaland [56].


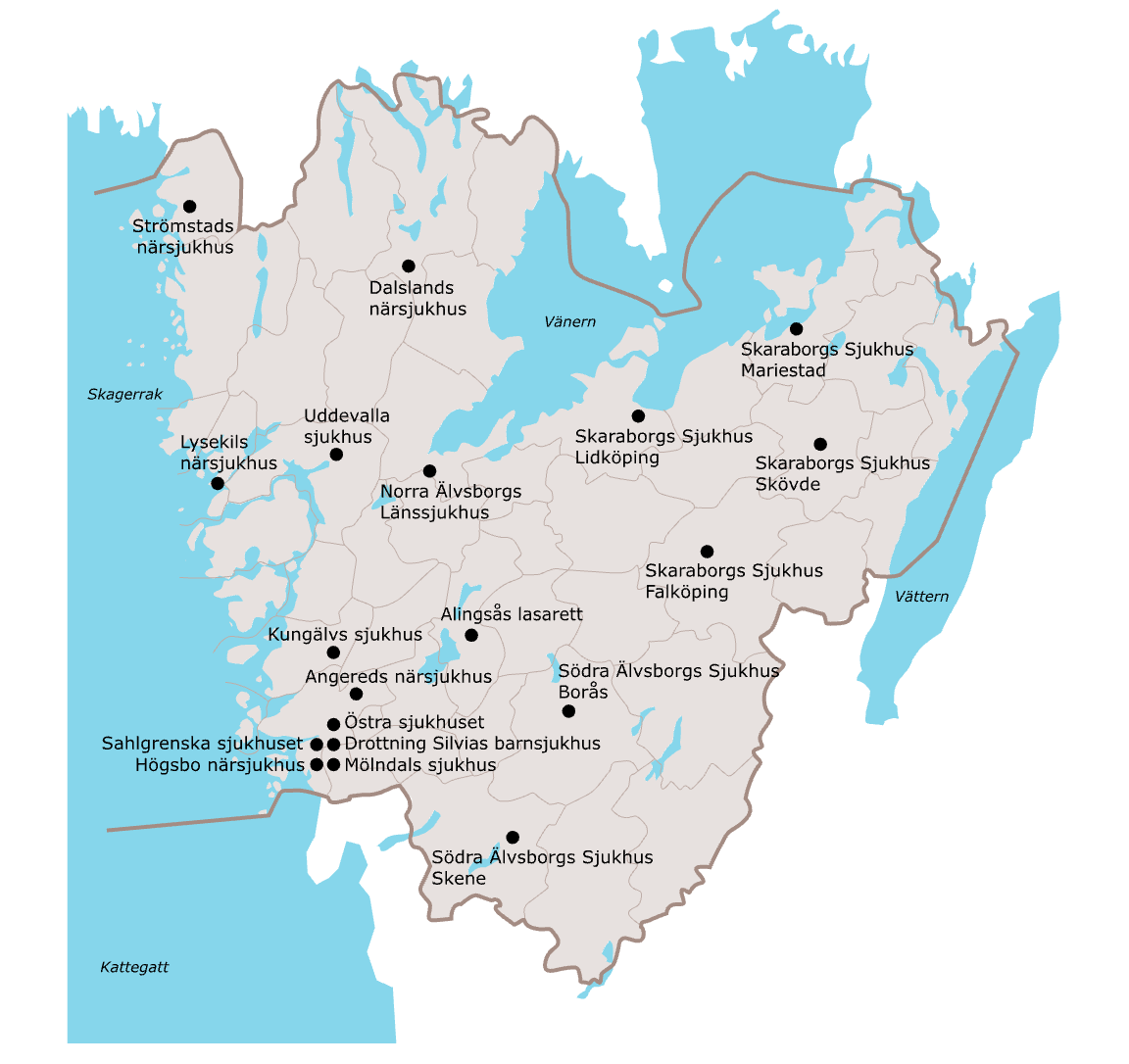

Supplement: Multimedia Appendix 2 [file diabetes-v11-e83287-s002.docx]

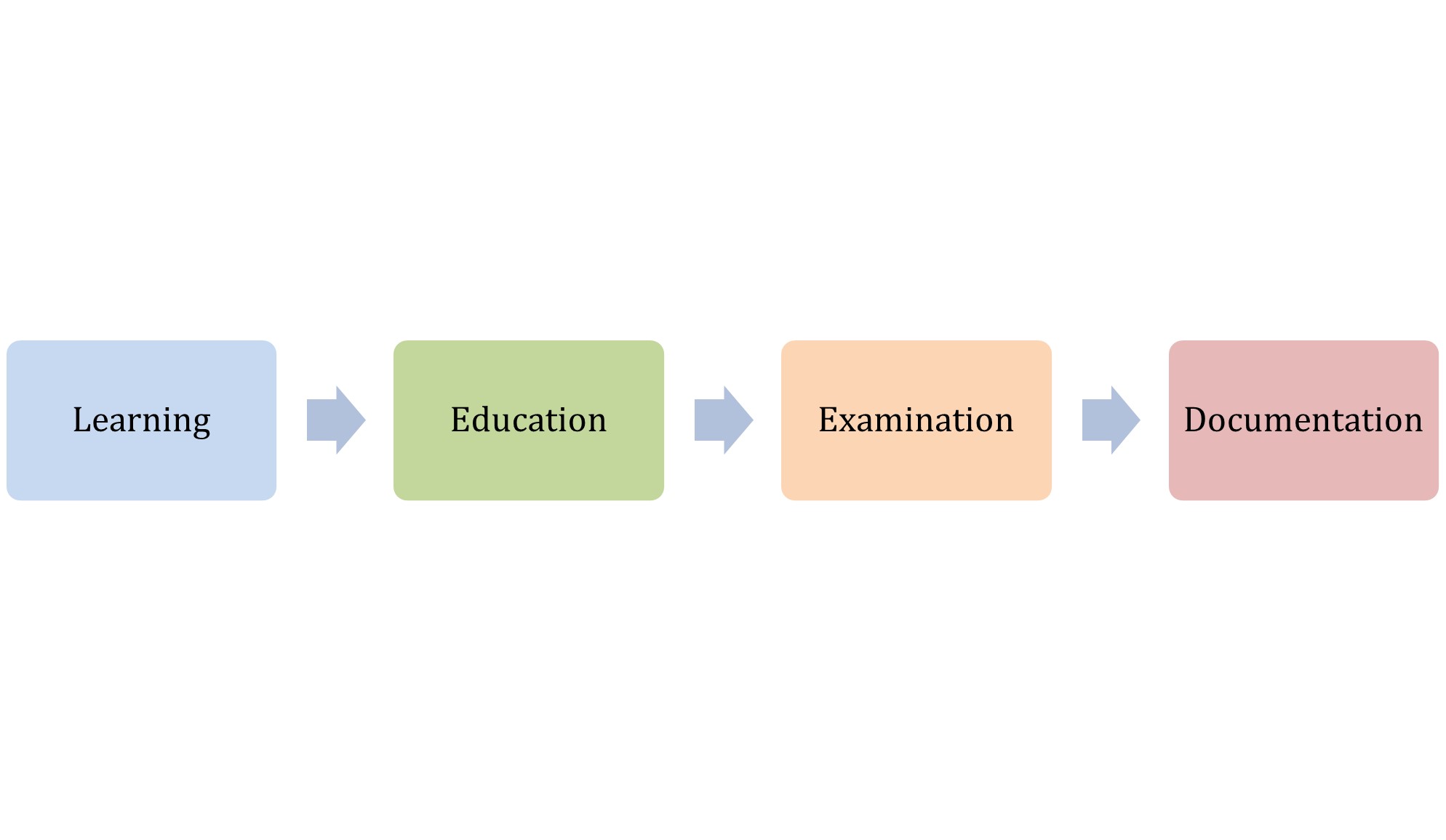

Supplement: Multimedia Appendix 4 [file diabetes-v11-e83287-s004.jpg]
