# Supplementary material for: User-Centered Development of a Digital Health Service for Diabetic Foot Ulcer Risk Stratification: Usability Study
Source: JMIR Diabetes. 2026 Apr 30;11:e83287. doi: 10.2196/83287 (PMC13132532; doi:10.2196/83287)
Supplement: Multimedia Appendix 3 [file diabetes-v11-e83287-s003.docx]

## Appendix 3. Personas a fictive person living with diabetes and a foot ulcer.

This persona, a fictive person, lives with diabetes at risk to develop foot ulcer.


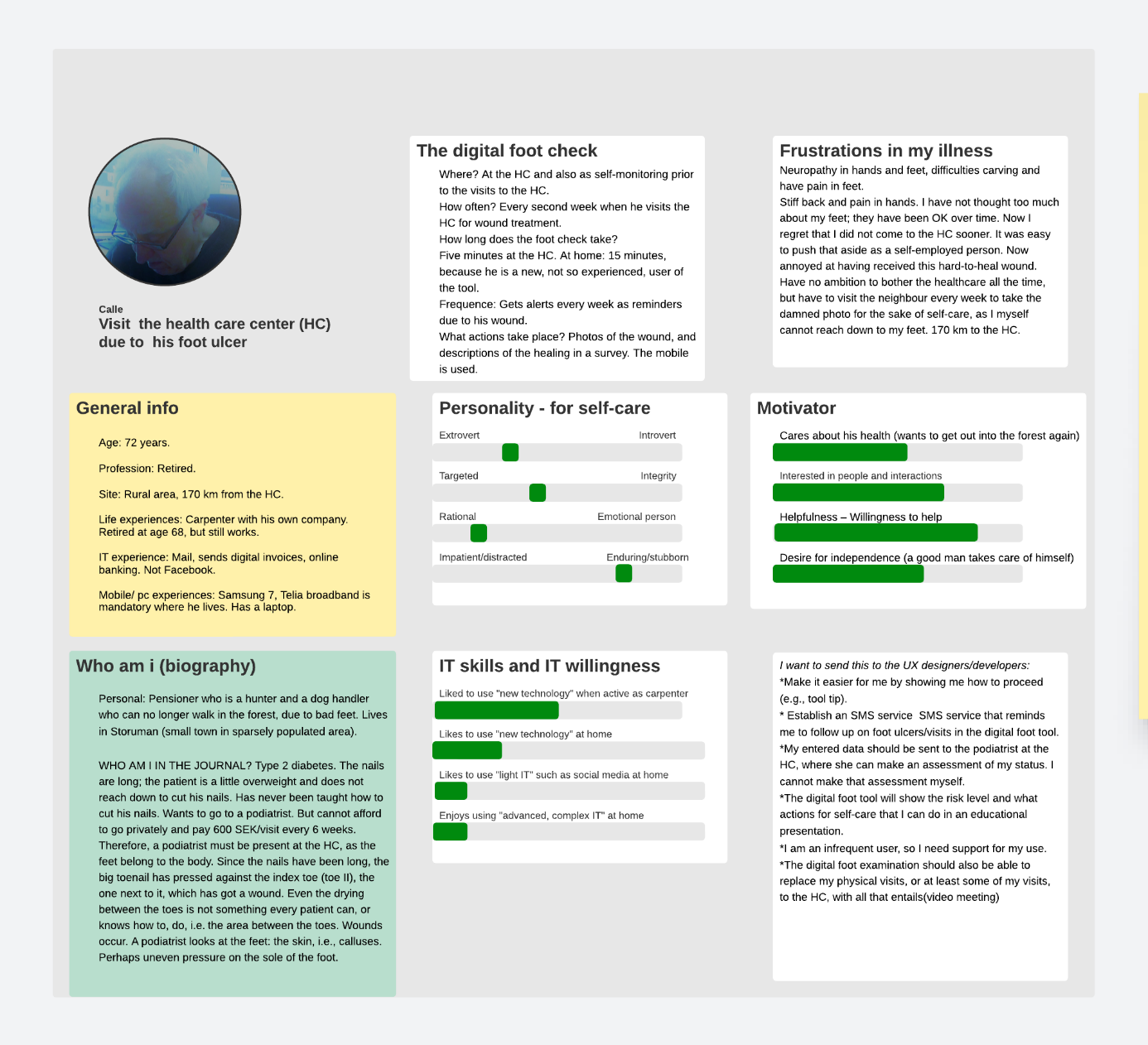


*Note: A persona is described according to a specific user group. Below is a summary of the main components in the context of using digital services for the persona Calle. The main components are:*

1. *Composition*
2. *Education and experience*
3. *Tools and aids*
4. *Used of intended tools*
5. *What, how often, how long, mandatory features and optional features for a given context of use.*
