# Supplementary material for: User-Centered Development of a Digital Health Service for Diabetic Foot Ulcer Risk Stratification: Usability Study
Source: JMIR Diabetes. 2026 Apr 30;11:e83287. doi: 10.2196/83287 (PMC13132532; doi:10.2196/83287)

## Appendix 5: Selected screenshots of CDSS workflow evaluated in the workshop


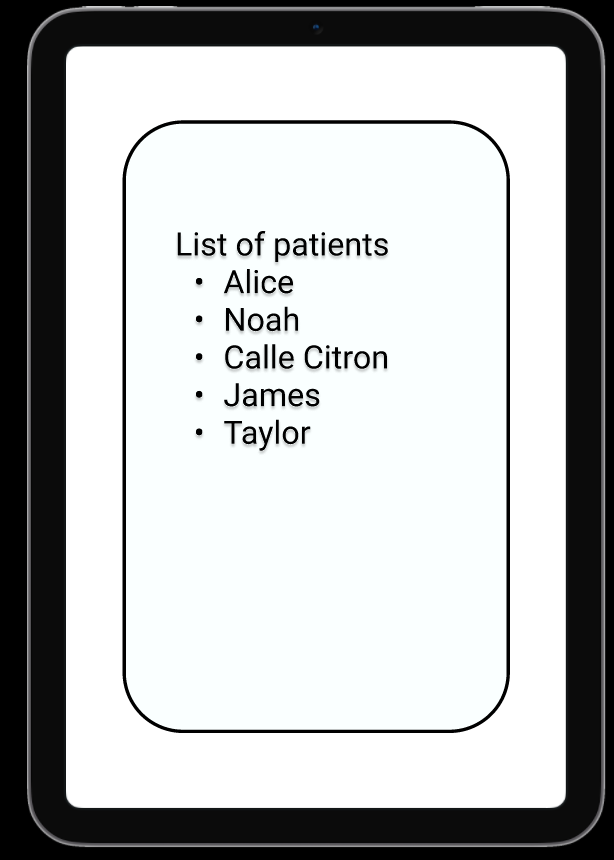
**Screenshot 1 – Main Interface: Patient Overview**


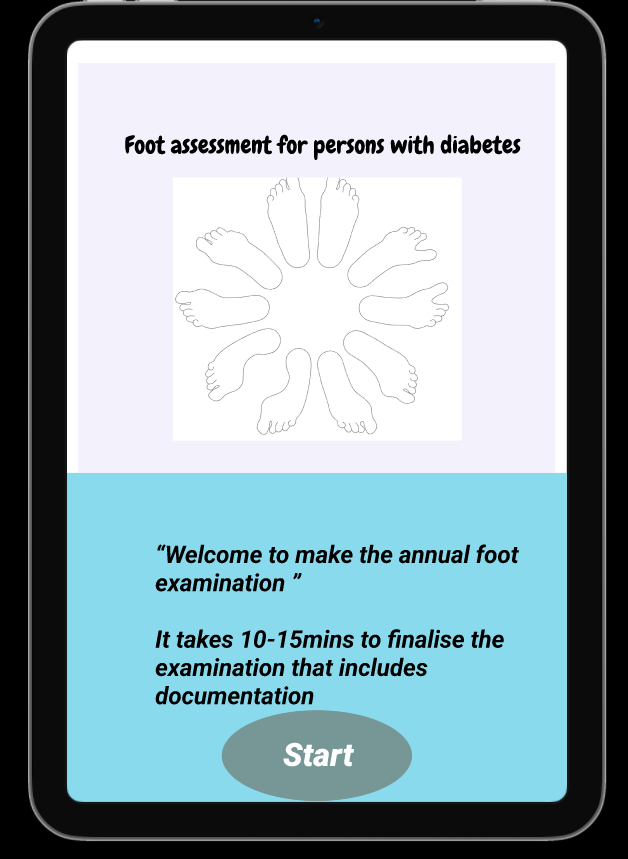


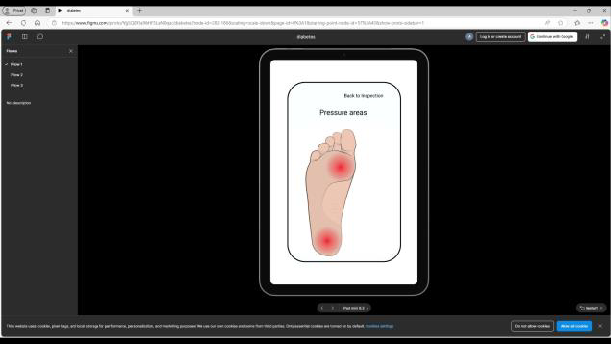

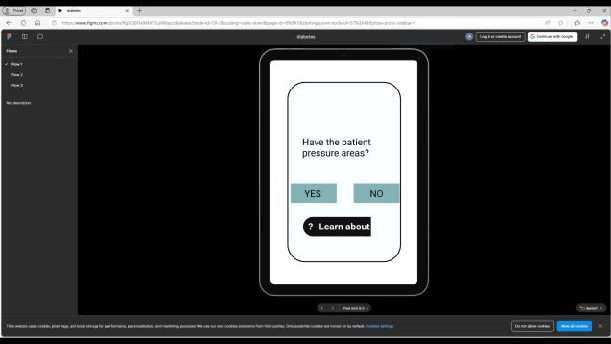
**Screenshot 2 – Skin Pathology Assessment Screen**


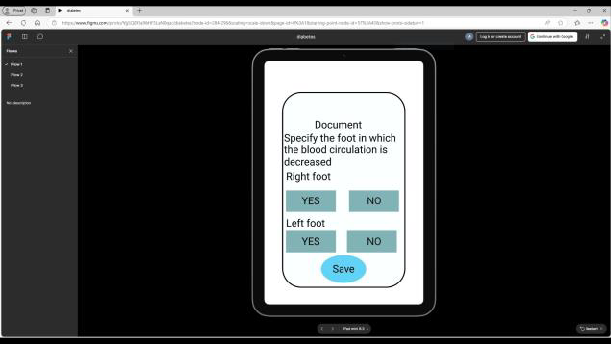

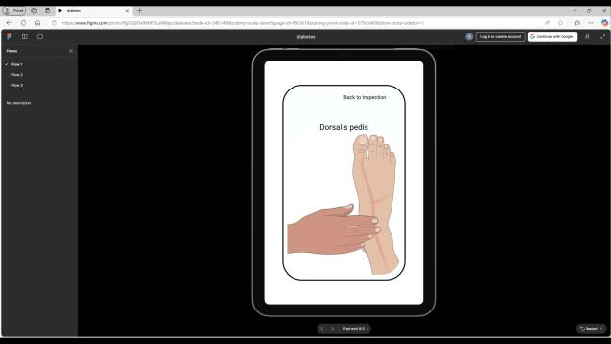

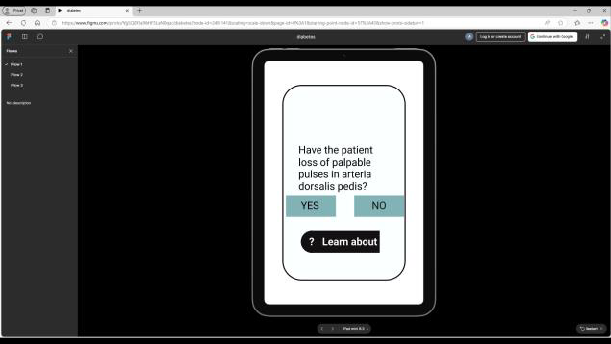
**Screenshot 3 – Circulation and Neuropathy Assessment**


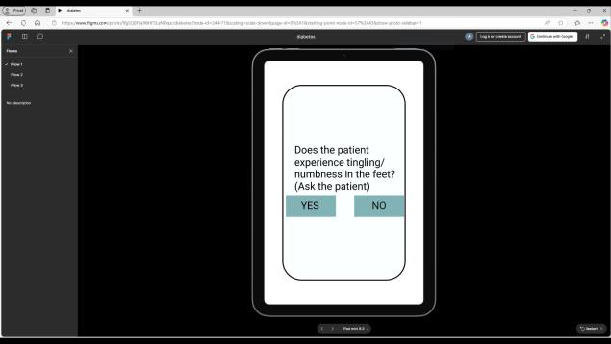
**Screenshot 4 – Neuropathy Assessment**


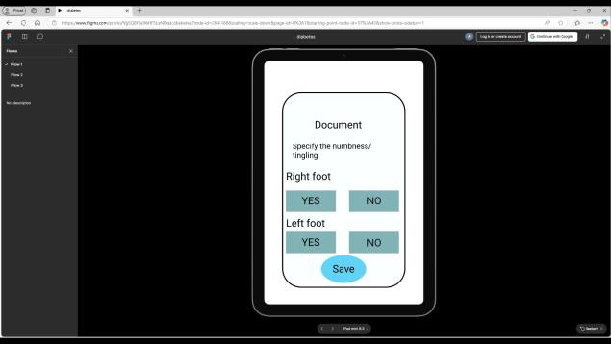

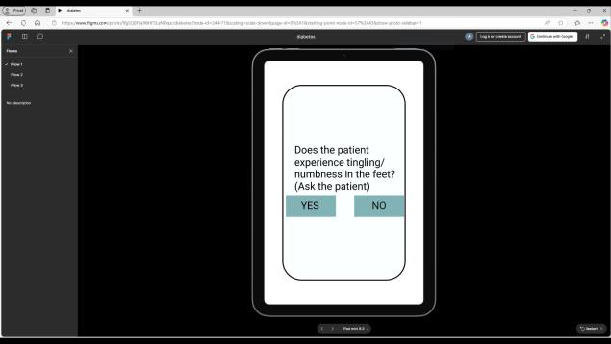

Supplement: Multimedia Appendix 5 [file diabetes-v11-e83287-s005.docx]
