# Supplementary material for: User-Centered Development of a Digital Health Service for Diabetic Foot Ulcer Risk Stratification: Usability Study
Source: JMIR Diabetes. 2026 Apr 30;11:e83287. doi: 10.2196/83287 (PMC13132532; doi:10.2196/83287)
Supplement: Multimedia Appendix 6 [file diabetes-v11-e83287-s006.docx]

# Appendix 6. Pre-test Survey

Participant ID: 11…….. Date: …………………..

Instructions: While completing this form, you are welcome to speak out loud if you have any comments or questions.

1. What is your name?

2. What year were you born?

3. Gender:
☐ Female ☐ Male ☐ Other

4. Where do you work?

5. What is your profession?

6. How many years have you worked with patients with diabetes?

7. Have you previously worked with digital tools to assess foot status in patients with diabetes?
☐ Yes ☐ No

8. If yes, which tools?

9. Have you previously used digital tools to assess health status in patients with diabetes (e.g. the patient questionnaire from the National Diabetes Register [NDR] or photo documentation)? (Please exclude the regular electronic medical record system.)
☐ Yes ☐ No

10. If yes, which tools?

11. Have you ever taken a photo of the foot as a basis for documentation?
☐ Yes ☐ No

12. Which tablet would you prefer to work with?
☐ Android ☐ iPad

Thank you for your participation!
