# Supplementary material for: User-Centered Development of a Digital Health Service for Diabetic Foot Ulcer Risk Stratification: Usability Study
Source: JMIR Diabetes. 2026 Apr 30;11:e83287. doi: 10.2196/83287 (PMC13132532; doi:10.2196/83287)
Supplement: Multimedia Appendix 7 [file diabetes-v11-e83287-s007.docx]

# Appendix 7. Post-test survey

Participant ID: …. Date: …………………..

Instructions: While completing this form, you are welcome to speak out loud if you have any comments or questions.

1. In the future, would you prefer to use a tablet or a mobile to make a digital foot examination?

2. Do you prefer to have an automatically generated risk scale in the app? Please, tell us about your thoughts about your answer.
☐ Yes ☐ No

3. In the future, would you find it useful to always register the results for the left and right foot separately? Please, tell us about your thoughts about your answer.
☐ Yes ☐ No

4. Do you think you would prefer a scroll-down function (going down on screen) to move to the next question in the foot examination instead of what you just tested?
☐ Yes ☐ No

5. How do you experience the structure of the app?

6. In the future, would you find it useful to always register the results for the left and right foot separately?
