# Supplementary material for: User-Centered Development of a Digital Health Service for Diabetic Foot Ulcer Risk Stratification: Usability Study
Source: JMIR Diabetes. 2026 Apr 30;11:e83287. doi: 10.2196/83287 (PMC13132532; doi:10.2196/83287)
Supplement: Checklist 1 [file diabetes-v11-e83287-s008.docx]

Supplementary. CONSORT-EHEALTH (V 1.6.1)

Note regarding completion of the CONSORT-EHEALTH checklist

The CONSORT-EHEALTH checklist was completed offline and compiled in this document. Attempts were made to complete the checklist using the official online submission form; however, repeated technical issues occurred during the process, including the save function not working and text fields disappearing after completion. As a result, previously entered responses were lost several times.

To ensure completeness and transparency, the checklist has therefore been completed manually in this document, following the structure and content of the original CONSORT-EHEALTH form. All questions are addressed in the same order as in the checklist, and responses have been kept concise in accordance with the intent of the reporting guideline.

*Ulla Hellstrand Tang*, on behalf of all co-authors, 2026-03-13

CONSORT-EHEALTH (V 1.6.1) - Submission/Publication Form

The CONSORT-EHEALTH checklist is intended for authors of randomized trials evaluating web-based and Internet-based applications/interventions, including mobile interventions, electronic games (incl multiplayer games), social media, certain telehealth applications, and other interactive and/or networked electronic applications. Some of the items (e.g. all subitems under item 5 - description of the intervention) may also be applicable for other study designs.

The goal of the CONSORT EHEALTH checklist and guideline is to be

1. a guide for reporting for authors of RCTs,
2. to form a basis for appraisal of an ehealth trial (in terms of validity)

CONSORT-EHEALTH items/subitems are MANDATORY reporting items for studies published in the Journal of Medical Internet Research and other journals / scientific societies endorsing the checklist.

Items numbered 1., 2., 3., 4a., 4b etc are original CONSORT or CONSORT-NPT (non-pharmacologic treatment) items.

Items with Roman numerals (i., ii, iii, iv etc.) are CONSORT-EHEALTH extensions/clarifications.

As the CONSORT-EHEALTH checklist is still considered in a formative stage, we would ask that you also RATE ON A SCALE OF 1-5 how important/useful you feel each item is FOR THE PURPOSE OF THE CHECKLIST and reporting guideline (optional).

Mandatory reporting items are marked with a red *.

In the textboxes, either copy & paste the relevant sections from your manuscript into this form - please include any quotes from your manuscript in QUOTATION MARKS, or answer directly by providing additional information not in the manuscript, or elaborating on why the item was not relevant for this study.

YOUR ANSWERS WILL BE PUBLISHED AS A SUPPLEMENTARY FILE TO YOUR PUBLICATION IN JMIR AND ARE CONSIDERED PART OF YOUR PUBLICATION (IF ACCEPTED).

Citation Suggestion (if you append the pdf as Appendix we suggest to cite this paper in the caption):

Eysenbach G, CONSORT-EHEALTH Group

CONSORT-EHEALTH: Improving and Standardizing Evaluation Reports of Web-based and Mobile Health Interventions

J Med Internet Res 2011;13(4):e126

URL: http://www.jmir.org/2011/4/e126/

doi: 10.2196/jmir.1923

PMID: 22209829

## Your name*First Last

Ulla Hellstrand Tang

## Primary Affiliation (short), City, Country*

University of Gothenburg

## Your e-mail address*

[ulla.tang@vgregion.se](mailto:ulla.tang@vgregion.se)

## Title of your manuscript*

User-centred development of a digital health service for diabetic foot ulcer risk stratification: usability study

## Name of your App/Software/Intervention*

D-Foot for Healthcare Professionals

## Language(s)*

English

## Accessibility

Intervention no longer accessible. The usability study is completed.

## Primary Medical Indication/Disease/Condition*

Diabetes

## Article Preparation Status/Stage *

Yes, submitted to a journal and after receiving initial reviewer comments submitted to a journal and accepted, but not published yet

## Journal *

JMIR Diabetes

## Manuscript tracking number *

JD ms#83287

TITLE AND ABSTRACT

## 1a. Identification as a randomized trial in the title. Does your paper address CONSORT item 1a?

Not applicable

## 1a-i. Identify the mode of delivery in the title . Does your paper address subitem 1a-i?

Yes. The term “digital health service” clearly indicates that the intervention is delivered digitally (i.e., via a digital platform/application).

## 1a-ii. Non-web-based components or important co-interventions in title. Does your paper address subitem 1a-ii?

Not applicable. The manuscript title is: “User-centred development of a digital health service for diabetic foot ulcer risk stratification: usability study.”The intervention evaluated was a digital health service incorporating a clinical decision support system (CDSS). There were no additional non-web-based components (e.g., telephone support, face-to-face therapy as part of the intervention, blended treatment elements) that functioned as co-interventions requiring mention in the title. The usability testing sessions were conducted in person; however, this was part of the research evaluation process and not part of the intervention itself. Therefore, no non-web-based components or co-interventions needed to be specified in the title.

## 1a-iii. Primary condition or target group in the title. Does your paper address subitem 1a-iii?

Yes. The title specifies the target condition: diabetic foot ulcer risk stratification.

## 1b. ABSTRACT: Structured summary of trial design, methods, results, and conclusions

Yes. Abstract summarizes development and usability evaluation of a clinician-facing clinical decision support system (CDSS).

## 1b-i. Key features/functionalities/components of the intervention and comparator in the METHODS section of the ABSTRACT. Does your paper address subitem 1b-i?

Yes. The Methods section of the abstract specifies that a clinical decision support system (CDSS) for diabetic foot ulcer (DFU) risk assessment was developed based on principles of human–computer interaction and designed to support foot assessment and risk stratification by healthcare professionals. This identifies the core function and intended use of the intervention. As this is a usability study without a comparator, no comparison intervention is applicable.

## 1b-ii. Level of human involvement in the METHODS section of the ABSTRACT. Does your paper address subitem 1b-ii?

Yes. The abstract specifies the level of human involvement by stating that the clinical decision support system (CDSS) is “to be used by healthcare professionals (HCPs) in foot assessment and risk stratification” and that HCPs evaluated the system in the usability testing. The intervention is healthcare professional–assisted (not fully automated) and is designed to support clinical decision-making rather than replace clinician involvement.

## 1b-iii. Open vs. closed, web-based (self-assessment) vs. face-to-face assessments in the METHODS section of the ABSTRACT. Does your paper address subitem 1b-iii?

Yes. The abstract specifies that the participants were healthcare professionals from Region Västra Götaland in Sweden, describing recruitment from a defined clinical setting rather than open web-based enrolment. This implies a closed, offline recruitment process rather than an open-access web trial.

The study is described as a mixed-methods usability testing approach, and usability outcomes were assessed using the System Usability Scale (SUS), a standardized self-reported questionnaire. As this is a usability study of a CDSS used by healthcare professionals, it is not a web-based trial with open enrolment, nor is blinding applicable.

## 1b-iv. Question: RESULTS section in abstract must contain use data

Abstract reports that result from the nine testers regarding usability/task metrics; no longitudinal uptake metrics apply in single-session testing.

## 1b-iv) RESULTS section in abstract must contain use data. Does your paper address subitem 1b-iv?

The Results section of the abstract reports:

• Post-test SUS scores (mean and SD)

• Mean difference and P value

• Task completion success rates (e.g., 7 out of 9 tasks; 78%)

• Number of testers achieving goals (e.g., 5 of 9 testers; 56%)

• Task completion times (efficiency data)

The number of testers (n=9) is added in the RESULTS and presented as “A total of nine participants participated“. Regarding use/uptake data, this is a structured usability study rather than a longitudinal web-based intervention. Therefore, metrics such as attrition, adherence over time, number of logins, or sustained use are not applicable. Instead, task completion rates and efficiency measures serve as appropriate “use data” within the context of usability testing.

## 1b-v. CONCLUSIONS/DISCUSSION in abstract for negative trials. Does your paper address subitem 1b-v?

## Yes, the abstract reports that post-test SUS scores showed a non-significant reduction (P = .071), but usability remained near the threshold of 70, interpreted as above average. The conclusion acknowledges that user expectations were higher than experienced usability and recommends further development and validation.

## 2a. In INTRODUCTION: Scientiﬁc background and explanation of rationale

Yes. Background explains DFU burden and need for structured guideline-based CDSS within clinical workflows.

## 2a-i. 2a-i) Problem and the type of system/solution. Does your paper address subitem 2a-i? *

Yes. Subitem 2a-i is addressed. The Introduction explains the clinical problem, the type of digital system developed, its intended users, and its role within the healthcare system.

## 2a-ii. Question: Scientiﬁc background, rationale: What is known about the (type of) system. Does your paper address subitem 2a-ii? *

Yes. The rationale is grounded in Human–Computer Interaction (HCI), ISO 9241-11 usability principles, and user-centred design. The CDSS integrates guideline-based DFU risk assessment into clinical workflow to improve usability, documentation, and consistency. The Introduction also reviews limitations of existing DFU digital tools, highlighting the need for a structured, systematically developed and evaluated CDSS.

## 2b. Question: In INTRODUCTION: Speciﬁc objectives or hypotheses. Does your paper address CONSORT subitem 2b? *

Yes. The manuscript states the study aim and specific research questions in the Introduction under the section “Aim & research question”: “The aim was to develop and evaluate a clinical decision support system (CDSS) to be used by healthcare professionals (HCPs) in foot assessment and risk stratification…”**.** The specific research questions are explicitly listed. Although formal hypotheses were not stated (appropriate for a formative usability study), the objectives and research questions are clearly defined and aligned with the study design.

## 3a. Question: Description of trial design (such as parallel, factorial) including allocation ratio

Partially applicable. Study is single-group mixed-method usability evaluation; no allocation ratio.

## METHODS

## 3a) Description of trial design (such as parallel, factorial) including allocation ratio. Does your paper address CONSORT subitem 3a? *

Yes (partially applicable). The manuscript describes the study design as a single-group mixed-methods usability study evaluating a CDSS prototype. As this was not a randomized trial, there was no allocation sequence or allocation ratio.

## 3b. Important changes to methods after trial commencement (such as eligibility criteria), with reasons. Does your paper address CONSORT subitem 3b? *

Yes (no changes). No important method changes after commencement are reported.

## 3b-i. Does your paper address subitem 3b-i?

Yes (no major changes occurred). The manuscript does not report any substantial bug fixes, system downtimes, content modifications, or functional changes during the usability testing phase because no major modifications were implemented that altered the intervention’s core functionality during the study period. This was a controlled usability evaluation of a defined CDSS prototype rather than a live, continuously evolving deployment. Minor iterative refinements resulting from usability feedback were part of the development process but did not constitute methodological changes affecting study design, data collection, or outcome interpretation. No unexpected events (e.g., technical failures, staffing changes, or system interruptions) occurred that influenced the conduct of the study.

## 4a. Eligibility criteria for participants. Does your paper address CONSORT subitem 4a? *

Yes. The manuscript specifies the target users and inclusion context. As this is a usability study rather than a randomized controlled trial, eligibility criteria were focused on professional background (e.g., nurses, podiatrists, physicians involved in diabetic foot care) rather than patient-level clinical characteristics. Therefore, eligibility criteria relevant to the study design are described and appropriate to the scope of the research.

## 4a-i. Computer / Internet literacy. Does your paper address subitem 4a-i?

Yes, partially. The manuscript does not explicitly state computer or internet literacy as a formal eligibility criterion. However, participants were healthcare professionals (HCPs) actively working within a Swedish healthcare region and routinely using digital systems in clinical practice. As stated: “Users, HCPs from Region Västra Götaland in Sweden evaluated the functions regarding effectiveness, efficiency and satisfaction using a mixed-methods usability testing approach.”. Given that the intervention is a clinical decision support system intended for integration into routine digital healthcare workflows, baseline digital literacy is inherent to the professional role of participating HCPs. Use of electronic medical records and digital tools is standard practice in Swedish healthcare settings. Therefore, while computer literacy was not explicitly listed as an inclusion criterion, it was implicitly ensured through the professional context and recruitment of practicing healthcare professionals.

## 4a-ii. Open vs. closed, web-based vs. face-to-face assessments. Does your paper address subitem 4a-ii? *

Yes. Closed recruitment from clinical settings; assessments were face-to-face workshops (not online self-enrolment). The study was conducted as a closed usability evaluation involving healthcare professionals recruited from Region Västra Götaland in Sweden. Participants were recruited within a defined clinical setting, not through open web-based enrolment. The study was not an open-access, self-enrolment web trial. It involved identified healthcare professionals testing a CDSS prototype in a structured usability setting. There was no anonymous or quasi-anonymous participation, and no risk of multiple identities, as participants were known professionals within the regional healthcare system.

## 4a-iii. Information giving during recruitment. Does your paper address subitem 4a-iii?

Yes. The manuscript describes that healthcare professionals from Region Västra Götaland participated in the usability evaluation of the CDSS. Participants were recruited within a defined clinical context and informed about the purpose of the study, namely, to evaluate the usability, effectiveness, efficiency, and satisfaction of the developed digital health service. As this was a usability study involving identified healthcare professionals rather than an open web-based trial with self-enrolment, recruitment occurred within a professional setting. Participants were informed about the study aim and procedures prior to participation and provided consent in accordance with ethical requirements. Because this was not an open-access web-based study, issues such as user self-selection driven by public advertisement or quasi-anonymous enrolment were not applicable. The structured recruitment within a professional healthcare context reduces risk of expectation bias related to promotional framing.

## 4. Settings and locations where the data were collected

Yes. Data were collected in hospital settings within Region Västra Götaland during structured workshops.

## 4b-i. Report if outcomes were (self-)assessed through online questionnaires

Yes. The manuscript specifies the setting and participant context. For example, the abstract states: “Users, HCPs from Region Västra Götaland in Sweden evaluated the functions regarding effectiveness, efficiency and satisfaction using a mixed-methods usability testing approach.” The Introduction further contextualises the study within Region Västra Götaland, Sweden, where the structured foot examination workflow was originally developed. s this was a usability evaluation of a CDSS prototype intended for clinical use, testing was conducted within a professional healthcare context rather than through open web-based participation.

## 4b-ii. Report how institutional aﬃliations are displayed, Does your paper address subitem 4b-ii?

Yes, where applicable. Institutional affiliations are clearly presented in the manuscript header. The corresponding author’s full institutional address and email are also provided. However, this was a closed usability study involving healthcare professionals recruited within Region Västra Götaland, not an open-access eHealth intervention promoted via public-facing media. Institutional affiliations were presented in the scientific manuscript as standard academic practice, but they were not used as recruitment material to influence volunteer enrolment. Participants were recruited within their professional healthcare context rather than through publicly branded eHealth platforms. Therefore, institutional display is unlikely to have influenced participation rates, engagement, or user reactions.

## 5. The interventions for each group with sufficient details to allow replication, including how and when they were actually administered

Yes. Intervention is described in sufficient detail to allow replication.

## 5-i. Mention names, credential, aﬃliations of the developers, sponsors, and owners. Does your paper address subitem 5-i?

Yes. The manuscript reports:

• Developers (named authors)

• Their institutional affiliations

• Funding sources

• Conflict of interest statement

• Role of the research team in development

## 5-ii. Describe the history/development process. Does your paper address subitem 5-ii?

Yes. The manuscript describes the historical background and iterative development process of the digital health service. The development builds on prior structured work as described in the paper. The Methods section describes a structured, iterative development approach: “The methodology comprised three phase (1) design, (2) testing, and (3) iterative refinement, following established usability testing principles…”

Conceptual development tools are also reported and stated as: “The software LucidChart… was used to create the conceptual design…” and “In the development… the software Figma… was used…”

The manuscript further describes structured usability testing and refinement: “The usability testing followed a structured, sequential process…”

The manuscript clearly describes:

• The historical origin of the intervention (paper-based workflow)

• Prior formative validation (workshops, qualitative evaluation)

• Theoretical grounding (HCI, user-centred design)

• Iterative development (design → testing → refinement)

• Tools used in development (LucidChart, Figma)

• Usability evaluation process

## 5-iii. Revisions and updating. Does your paper address subitem 5-iii?

Yes, partially. The manuscript describes a structured, phased development process: “The methodology comprised three phases: (1) design, (2) testing, and (3) iterative refinement, following established usability testing principles…” .Usability testing was conducted during 2022 in defined workshop sessions: “Usability testing sessions were conducted at these hospital sites during 2022.” The CDSS evaluated during the workshops was a defined prototype developed using LucidChart and Figma.

The manuscript does not report a formal version number of the prototype. However:

• The intervention was functionally stable during the usability testing sessions.

• No major revisions, feature additions, or content changes were implemented during data collection.

• Identified improvements (e.g., navigation, automated risk scoring, language adjustments) are reported as post-test refinement recommendations, not mid-trial updates.

The system did not include dynamic components such as live data feeds, adaptive content, or continuously updated modules that would affect replicability. Because this was a controlled formative usability evaluation of a prototype, the intervention content was effectively unchanged during the testing period.

## 5-iv. Quality assurance methods. Does your paper address subitem 5-iv?

Yes. The manuscript describes several measures ensuring the accuracy, reliability, and quality of the content embedded in the digital health service. First, the intervention is grounded in national and international clinical guidelines. The usability testing incorporated systematic evaluation methods (think-aloud protocol, SUS, task-based testing) to identify inaccuracies, ambiguities, and areas needing refinement.

Yes, subitem 5-iv is addressed, as described in the paper:

• Content is guideline-based and evidence-informed

• Face validity was secured through professional workshops

• Structured workflows ensure standardisation

• Systematic usability testing supported refinement

## 5-v. Ensure replicability by publishing the source code, and/or providing screenshots/screen-capture video, and/or providing ﬂowcharts of the algorithms. Does your paper address subitem 5-v?

Partially. The manuscript provides materials supporting replicability, including structured workflow documentation (Appendix 1), screenshots of the CDSS (Appendix 6), the guideline-based risk stratification model (Figure 1), and descriptions of development tools (LucidChart and Figma).

Although the source code is not published, the workflow description, risk logic, screenshots, and guideline references provide sufficient transparency for conceptual replication in this formative usability study of a prototype CDSS.

## 5-vi) Digital preservation. Does your paper address subitem 5-vi?

Yes, partially. No public URL/archiving is provided; screenshots and documentation support preservation.

## 5-vii. Access. Does your paper address subitem 5-vii?

Yes (study context). The paper clarifies:

• Access occurred in a controlled clinical environment.

• Participants did not self-enroll online.

• No payment was required to access the system.

• Participants were members of a defined professional group (HCPs within VGR).

• Devices (tablets) were provided during the workshops.

• Internet/platform access was managed within the hospital setting.

Because this was a prototype evaluated in a controlled usability context (not a publicly available platform), open access, membership fees, or login distribution to the public were not applicable. A public demo mode or reviewer login is not currently available, as the system is not yet deployed as a live product. Subitem 5-vii is adequately addressed. The manuscript clearly reports:

• Who had access

• Where and how access occurred

• That access was controlled and workshop-based

• That no public or paid access was involved

## 5-viii. Mode of delivery, features/functionalities/components of the intervention. Does your paper address subitem 5-viii?

Yes. The intervention is a tablet-based CDSS used by healthcare professionals during structured usability workshops. It includes education, examination, and documentation modules, guideline-based DFU risk stratification, and structured registration of foot findings. The system builds on national clinical guidelines and a validated paper-based workflow. Screenshots are provided (Appendix 6). The design is grounded in Human–Computer Interaction (HCI), ISO 9241-11 usability principles, and user-centred design. No comparator intervention was included, as this was a formative usability study.

## 5-ix. Describe use parameters. Does your paper address subitem 5-ix?

Yes. The intervention was used in structured, task-based usability sessions reflecting routine diabetes foot care workflows. Each session lasted approximately two hours and involved predefined simulated clinical tasks performed by HCPs. The system was not used freely or independently by participants. As this was a formative usability study of a prototype, long-term use or dose-response effects were not assessed.

## 5-x. Clarify the level of human involvement. Does your paper address subitem 5-x?

Yes. Human involvement is central: HCPs used CDSS; researchers facilitated only the test sessions. The manuscript states that the CDSS is intended for use by healthcare professionals and that the intervention requires active professional use (not automated or patient-only).

## 5-xi. Report any prompts/reminders used. Does your paper address subitem 5-xi?

Not applicable. No prompts/reminders were used. This study was a structured, workshop-based usability evaluation of a prototype CDSS conducted in hospital settings. Participants attended scheduled usability sessions lasting approximately two hours. Because:

• The intervention was not deployed longitudinally,

• Participants did not access the system independently over time,

• There was no home-based or ad libitum use,

• The system did not include automated notifications, reminders, emails, SMS, or push prompts or reminders were used to encourage continued engagement.

The CDSS prototype evaluated in this study does not include built-in reminder functions, and no external prompting mechanisms (letters, emails, SMS, phone calls) were part of the intervention or study protocol. As this was a single-session usability evaluation rather than a behaviour change intervention or longitudinal trial, reminder mechanisms were not applicable.

## 5-xii- Describe any co-interventions (incl. training/support). Does your paper address subitem 5-xii?

Yes. Structured introduction/instructions/observation described; no additional clinical co-interventions were delivered. The manuscript describes the structured support during usability testing. The session was introduced by study team members, observers were present using the think-aloud method, and participants were provided with tablets and written instructions: “The usability test was introduced by two persons from the study team (SR and UT).” And “The observers (n=2) made observations…” . And “The testers are provided with tablets and documents…”

No formal training program or additional clinical co-intervention was provided. The CDSS is intended for independent use by trained healthcare professionals in routine care. The manuscript also notes future onboarding needs: “Future iterations of the CDSS should incorporate… enhanced onboarding and training modules…”

## 6a. Completely deﬁned pre-speciﬁed primary and secondary outcome measures, including how and when they were assessed. Does your paper address CONSORT subitem 6a?

Yes. Pre-specified outcomes were: SUS (pre/post), task effectiveness (completion), task efficiency (time), and qualitative feedback. The manuscript defines outcomes and their timing. Usability was assessed with pre- and post-test SUS. Effectiveness was measured as task completion: “Task effectiveness was evaluated by calculating the proportion of participants who successfully completed each task.” Efficiency was measured as time-on-task: “Task efficiency was assessed by measuring the time required to complete each predefined task…”

Assessments occurred during structured sessions lasting approximately two hours, with qualitative data collected via think-aloud and open-ended surveys. Outcomes were pre-specified and aligned with the study aims (effectiveness, efficiency, satisfaction). Subitem 6a is adequately addressed. The manuscript defines:

• What was measured

• How it was measured

• When it was measured

• The analytic approach used

## 6a-i. Online questionnaires: describe if they were validated for online use and apply CHERRIES items to describe how the questionnaires were designed/deployed. Does your paper address subitem 6a-i?

Subitem 6a-i is not applicable. The validated SUS instrument was used, but the questionnaires were administered within a controlled in-person usability testing context and not as an online e-survey environment. Therefore, CHERRIES reporting requirements were not applicable.

## 6a-ii. Describe whether and how “use” (including intensity of use/dosage) was deﬁned/measured/monitored. Does your paper address subitem 6a-ii?

Yes. “Use” measured as task completion rates and time-on-task within the session; no logins/logfiles. “Use” was defined as participation in a structured two-hour usability session and completion of predefined clinical tasks. It was measured through task completion rates (effectiveness) and time-on-task (efficiency), rather than logins or longitudinal exposure metrics.

6a-iii. Describe whether, how, and when qualitative feedback from participants was defined/measured/monitored. Does your paper address subitem 6a-iii?

Yes. Qualitative feedback obtained via think-aloud, observation notes, and open-ended survey responses. The manuscript describes when, how, and by which methods qualitative feedback was collected. Feedback was obtained during and after structured usability sessions. Qualitative data were analysed using inductive content analysis.

## 6b. Any changes to trial outcomes after the trial commenced, with reasons. Does your paper address subitem 6a-iii? Does your paper address subitem 6b?

Yes (no changes). No changes to outcomes after commencement are reported. The manuscript does not report any changes to predefined outcomes after trial commencement. Outcomes (SUS, task effectiveness, task efficiency, qualitative feedback) align with the stated aim: “The aim was to develop and evaluate a clinical decision support system (CDSS)… Users… evaluated the functions regarding effectiveness, efficiency and satisfaction…”

The study was prospectively registered: “ClinicalTrials.gov ID: NCT05692778”. No outcomes were added, modified, or dropped after data collection began.

## 7a. How sample size was determined NPT: When applicable, details of whether and how the clustering by care provides or centers was addressed. Does your paper address CONSORT subitem 7a?

Yes, partly applicable. Sample size justified by usability methodology (diverse roles; diminishing returns beyond ~5–10). The sample size was determined based on established usability testing methodology rather than statistical power calculations. Clustering by care providers or centres was not applicable, as this was not a cluster-randomised or comparative trial.

## 7a-i. Describe whether and how expected attrition was taken into account when calculating the sample size. Does your paper address CONSORT subitem 7a-i?

Not applicable. No attrition expected in single-session design; not used for sample size calculation.

## 7b. When applicable, explanation of any interim analyses and stopping guidelines. Does your paper address CONSORT subitem 7b?

Not applicable. No interim analyses or stopping guidelines were used.

## 8a. Method used to generate the random allocation sequence NPT: When applicable, how care providers were allocated to each trial group, Does your paper address CONSORT subitem 0a?

Not applicable. This was not a randomized controlled study.

## 8b. Type of randomisation; details of any restriction (such as blocking and block. Does your paper address CONSORT subitem 8b?

Not applicable. This was not a randomized controlled study.

## 9. Mechanism used to implement the random allocation sequence (such as sequentially numbered containers), describing any steps taken to conceal the sequence until interventions were assigned. Does your paper address CONSORT item 9?

Not applicable. This was not a randomized controlled study.

## 10. Who generated the random allocation sequence, who enrolled participants, and who assigned participants to interventions. Does your paper address CONSORT item 10?

Not applicable. This was not a randomized controlled study.

## 11a. If done, who was blinded after assignment to interventions (for example, participants, care providers, those assessing outcomes) and how. Does your paper address CONSORT item 11a?

Not applicable. No blinding of participants/personnel.

## 11a-i. Specify who was blinded, and who wasn’t. Does your paper address CONSORT item 11a-i?

Not applicable. No blinding of participants/personnel.

## 11a-ii. Discuss e.g., whether participants knew which intervention was the “intervention of interest” and which one was the “comparator”. Does your paper address CONSORT item 11a-ii?

Not applicable. No blinding of outcome assessment.

## 11b. If relevant, description of the similarity of interventions (this item is usually not relevant for ehealth trials as it refers to similarity of a placebo or sham intervention to a active medication/intervention). Does your paper address CONSORT item 11b?

Not applicable. No similarity of interventions to mask allocation (no groups).

## 12a. Statistical methods used to compare groups for primary and secondary. Does your paper address CONSORT item 12a?

Partially applicable. Descriptive statistics and paired t-test for pre–post SUS are described; no between-group comparisons.

## 12a-i. Imputation techniques to deal with attrition / missing values. Does your paper address CONSORT item 12a-i?

Not applicable. No attrition; no imputation needed.

## 12b. Methods for additional analyses, such as subgroup analyses and adjusted analyses. Does your paper address CONSORT item 12b?

Not applicable. No additional subgroup/adjusted analyses.

## 13a. For each group, the numbers of participants who were randomly assigned, received intended treatment, and were analysed for the primary outcome. Does your paper address CONSORT item 13a?

Not applicable to randomisation flow. All nine enrolled participants received the test and were analysed.

## 13b. For each group, losses and exclusions after randomisation, together with reasons. Does your paper address CONSORT item 13a?

Not applicable. No loss to follow-up/attrition in single-session design.

## 13b-i. Attrition diagram. Does your paper address CONSORT item 13b-i?

Not applicable. No attrition diagram required (0 dropout).

## 14a. Dates deﬁning the periods of recruitment and follow-up. Does your paper address CONSORT item 14a?

Yes. Recruitment/testing period reported (2022). Yes (recruitment period reported; no follow-up period applicable). The manuscript specifies when usability testing was conducted. Because this was a single-session formative usability study, there was:

• No longitudinal follow-up

• No repeated measurement period

• No extended trial phase

Each participant attended a defined usability session lasting approximately two hours

## 14a-i. Indicate if critical “secular events” fell into the study period. Does your paper address subitem 14a-i?

Not applicable. No critical secular events affecting technology/resources are reported.

## 14b. Why the trial ended or was stopped (early). Does your paper address subitem 14b?

Not applicable. Study ended as planned; not stopped early.

## 15. A table showing baseline demographic and clinical characteristics for each group. Does your paper address subitem 15?

Yes (descriptive). Baseline/professional characteristics of participants are reported; no group table needed.

## 15-i. Report demographics associated with digital divide issues. Does your paper address subitem 15-i?

Yes. Age/profession/experience and prior digital tool use reported (digital divide–relevant characteristics). Yes, within the context of a professional HCP sample. The manuscript reports several characteristics relevant to digital divide considerations: age, professional background and expertise, digital literacy and prior exposure and device preferences. This study involved healthcare professionals working within a Swedish public healthcare system, not members of the general population. Therefore:

• Socioeconomic status is less relevant.

• Internet access was ensured within clinical settings.

• Baseline professional digital exposure is inherent to their role.

Digital literacy differences were partially captured through reported prior use of digital tools and variation in device familiarity.

## 16. For each group, number of participants (denominator) included in each analysis and whether the analysis was by original assigned groups. Does your paper address item 16?

Yes (single denominator). All analyses used n=9 participants; no group allocation.

## 16-i. Report multiple “denominators” and provide deﬁnitions. Does your paper address subitem 16-i?

Not applicable. Single session; no multiple exposure denominators (e.g., logins/weeks).

## 16-ii. Primary analysis should be intent-to-treat. Does your paper address subitem 16-ii?

Not applicable. No randomisation; intent-to-treat framework not relevant.

## 16-iii. If analysing only “users” (or higher-dose users), clarify self-selection and bias; ensure interpretation reflects non-random sample. Does your paper address subitem 16-iii?

Not applicable. No user-only subgroup analyses; all participants were users in-session.

## 17a. For each primary and secondary outcome, results for each group, and the estimated effect size and its precision (such as 95% conﬁdence interval). Does your paper address item 17a?

Partially applicable. Outcomes reported with descriptive statistics; no between-group effect sizes/Confidence Interval needed.

## 17a-i. Presentation of process outcomes such as metrics of use and intensity of use.

Yes. Process outcomes include task success rates and time-on-task. “Use” was defined within a structured usability workshop ( approximately 2 hours) where HCPs completed predefined clinical tasks reflecting routine workflows. Time-on-task and task completion were directly observed and recorded. As this was a single-session usability evaluation, longitudinal exposure metrics (e.g., logins or duration of use) were not applicable.

## 17b. For binary outcomes, presentation of both absolute and relative effect sizes is recommended. Does your paper address CONSORT subitem 17b?

Not applicable. No group comparisons; binary task outcomes reported descriptively.

## 18. Results of any other analyses performed, including subgroup analyses and adjusted analyses, distinguishing pre-specified from exploratory. Does your paper address CONSORT item 18?

Not applicable. No subgroup or adjusted analyses performed.

## 18-i. Subgroup analysis of comparing only users. Does your paper address CONSORT item 18-i?

Not applicable. No user-only subgroup analysis; all participants used the system during testing.

## 19. All important harms or unintended effects in each group. Does your paper address CONSORT item 19?

Yes. No adverse events reported; low-risk professional usability study.

## 19-i. Include privacy breaches, technical problems. Does your paper address CONSORT subitem 19. Does your paper address subitem 19-i?

Yes. No privacy breaches or serious technical incidents occurred. The manuscript reports confidentiality safeguards and informed consent. Minor usability issues (e.g., screen sensitivity, overlooked interface elements, language clarity) were documented as usability findings. Participants also noted positive unintended effects, including potential improvements in equitable and person-centred DFU prevention.

## 19-ii. Include qualitative feedback from participants or observations from staff/researchers. Does your paper address subitem 19-ii?

Yes. Qualitative feedback was collected via think-aloud protocols, observations, and post-test surveys. Strengths and shortcomings (e.g., navigation issues, language challenges, workflow improvements) are reported in the Results and summarized in Table 2.

## 20. Trial limitations, addressing sources of potential bias, imprecision, and, if relevant, multiplicity of analyses. Does your paper address subitem 20?

Yes. Limitations discussed (sample size, context, usability constraints, language).

## 20-i. Typical limitations in ehealth trials. Does your paper address subitem 20-i?

Yes. Typical eHealth limitations addressed; blinding/multiplicity largely not applicable; usability issues and contextual biases discussed. The manuscript explicitly identifies limitations, unanswered questions, and directions for future research. Suggestion for future work was:

1. Need for larger and real-world validation studies

2. Implementation

3. Regulatory and validation requirements: “Further development must also align with regulatory requirements for medical devices… including clinical validation, usability engineering, and risk management…”

4. Algorithm validation and clinical accuracy: “Future studies should evaluate the accuracy, reliability, and adaptability of automated risk stratification algorithms…”

5. Integration and contextual adaptation: “Integration with electronic health records and quality registers… will be essential for sustainable implementation.”

## 21. Generalisability (external validity, applicability) of the trial findings. Does your paper address subitem 21?

Yes. External validity discussed; need for larger real-world implementation and broader user diversity.

## 21-i. Generalizability to other populations. Does your paper address subitem 21-i?

## Yes. The manuscript discusses generalisability beyond the regional sample and recommends further validation through larger real-world implementation studies. It highlights potential scalability across healthcare systems and countries while acknowledging limitations due to the small HCP sample and controlled workshop setting. The intervention targets healthcare professionals rather than the general Internet population

## 21-ii. Discuss if there were elements in the RCT that would be different in a routine different in a routine application setting. Does your paper address subitem 21-ii?

Not applicable. No comparator groups; unequal expertise between arms does not apply.

## 22. Interpretation consistent with results, balancing benefits and harms, and considering other relevant evidence. Does your paper address subitem 22?

Yes. The Discussion interprets findings consistently with the results (SUS scores, task completion variability, time-on-task) and relates them to existing usability and DFU decision-support literature. Benefits (structured workflow and potential for equitable care) are balanced with limitations (small sample, usability challenges, language issues). The study is framed as formative, with recommendations for further validation and real-world implementation.

## 22-i. Restate study questions and summarize the answers suggested by the data, starting with primary outcomes and process outcomes (use). Does your paper address subitem 22-i?

Yes. The manuscript:

• Restates the study aim and research questions

• Reports primary usability outcomes (SUS)

• Reports process outcomes (task effectiveness and efficiency)

• Summarizes how findings answer the research questions

• Provides a balanced synthesis in the Discussion and Conclusion

## 22-ii. Highlight unanswered new questions, suggest future research. Does your paper address subitem 22-ii?

Yes. The manuscript:

• Identifies unanswered questions (implementation, scalability, validation)

• Highlights methodological limitations

• Suggests future summative and effectiveness studies

• Recommends regulatory alignment and algorithm validation

• Emphasizes integration and contextual adaptation

## 23. Registration number and name of trial registry. Does your paper address CONSORT subitem 23?

Yes. Trial registration reported: ClinicalTrials.gov NCT05692778.

## 24. Where the full trial protocol can be accessed, if availableDoes your paper address CONSORT subitem 24?

Yes, partly. The ClinicalTrials.gov registration includes key protocol information such as study design, objectives, eligibility criteria, and outcome.

## 25. Sources of funding and other support (such as supply of drugs), role of funders. Does your paper address CONSORT subitem 25?

Yes. The manuscript clearly reports sources of funding: “The study was supported by the Innovation Platform in Region Västra Götaland, Gothenburg Diabetes Association, Stiftelsen Skobranschens Utvecklingsfond, Gunnar Holmgrens Minne, Felix Neuberghs Foundation.” The manuscript also includes a conflict of interest statement: “Conflict of interest: None declared.”

No commercial sponsor, pharmaceutical supply, or industry funding is reported. The funding sources are public or foundation-based and there is no indication that funders influenced study design, data collection, analysis, interpretation, or manuscript preparation.

## X26-i) Comment on ethics committee approval. Does your paper address subitem X26-i?

**Yes.** The manuscript reports:

- Ethics committee approval (with registration number)
- Compliance with the Helsinki Declaration
- Written informed consent procedures
- Consent for publication of identifiable materials

## X26-ii. Safety and security procedures. Does your paper address CONSORT subitem X26-ii?

Yes. Participants received oral and written information about the study and provided written informed consent. Consent for publication of identifiable images or data was also obtained. Confidentiality and the right to withdraw were explained. As this was an in-person usability study, consent was obtained offline using signed forms.

## X26-iii) Safety and security procedures. Does your paper address subitem X26-iii?

Yes. The manuscript addresses ethical safeguards, privacy protection, and measures to reduce potential harm. Safety for participants were secured by:

1. The study received formal ethical approval.

2. Minimising risk of harm. The study involved healthcare professionals performing simulated tasks in a controlled hospital setting. No real patient interventions were conducted during usability testing. The manuscript acknowledges potential inconvenience.

3. Data protection and security context: The CDSS was developed within a public healthcare-academic collaboration and designed to support structured documentation compatible with Electrial Health Recordss/Nationlal Diabetes Registry.

The usability testing was conducted in controlled clinical environments, and no patient-identifiable live clinical data were processed during the workshop sessions. There was no need for hotlines, adverse event monitoring, or harm-detection systems, as this was not a patient-facing or clinical effectiveness trial.

## X27. Conﬂicts of Interest (not a CONSORT item)

Yes. Conﬂicts of Interest statement included; developer–evaluator relationship discussed.

## X27-i. State the relation of the study team towards the system being evaluated. Does your paper address subitem X27-i?

Yes. The manuscript reports that members of the study team were involved in the development of the digital health service. For example: “An innovation team, consisting of a clinical researcher, a researcher in health informatics, a master student in biomedical engineering, and a researcher in biomedical engineering, formulated the strategy for the development of the digital health service.”

It also states: “Prior to this study (in 2020), the principal investigator (author UT) led a regional development group… [that] created a workflow for a foot examination…”

Thus, the authors were both developers and evaluators of the CDSS prototype. The manuscript also includes a formal declaration: “Conflict of interest: None declared.”

No commercial ownership, intellectual property claims, or financial interests are reported. The development occurred within an academic and public healthcare collaboration.

The manuscript transparently indicates that:

• Members of the study team were involved in the development of the CDSS.

• The same team conducted the usability evaluation.

• No financial conflicts of interest are declared.

• The intervention was developed within public healthcare and academic institutions.

## About the CONSORT EHEALTH checklist. As a result of using this checklist, did you make changes in your manuscript?

Yes, the number of participants in the study is clearly presented in the abstract.

## How much time did you spend on going through the checklist INCLUDING making changes in your?

24 hours.

## Author Comments on the CONSORT-EHEALTH Checklist Process

1. A maximum word limit for each question would be helpful to ensure concise and structured responses.

2. During completion of the online checklist, several technical difficulties occurred:
 - The save function did not work reliably.
 - Text fields disappeared after being completed.
 - Responses had to be re-entered multiple times.

3. Providing the option to download a working offline version (e.g., editable PDF or Word form) would facilitate completion and prevent data loss.
